# Characterization of TLR9 responsiveness in cell subsets derived from *in vitro* pDC differentiation of hematopoietic stem and progenitor cells

**DOI:** 10.3389/fimmu.2025.1550397

**Published:** 2025-03-27

**Authors:** Sabina Sánchez Hernández, Tobias Wang Bjerg, Ian Helstrup Nielsen, Anders Laustsen, Hai Q Tang, Lars Henning Pedersen, Eynav Klechevsky, Martin R. Jakobsen, Rasmus O. Bak

**Affiliations:** ^1^ Department of Biomedicine, Aarhus University, Aarhus, Denmark; ^2^ Department of Clinical Medicine, Aarhus University, Aarhus, Denmark; ^3^ Department of Obstetrics and Gynaecology, Aarhus University Hospital, Aarhus, Denmark; ^4^ Department of Pathology and Immunology, Division of Immunobiology, Washington University School of Medicine, St. Louis, MO, United States

**Keywords:** plasmacytoid dendritic cells, CD34 hematopoietic stem cells, *in vitro* differentiation, subsets, type I IFN

## Abstract

Plasmacytoid dendritic cells (pDCs) are multifunctional immune cells with roles in both the innate and adaptive immune system. Their hallmark function is production of large amounts of type I interferons in response to viral infections, but they are also capable of producing a range of other cytokines, antigen presentation, and cytotoxicity. Their potential as an immunotherapy for cancer and infectious disease is being explored, but broad application of these cells is challenged by low frequency in the blood and low viability during *ex vivo* culturing. We have previously developed an effective *in vitro* differentiation protocol for producing pDCs from CD34+ hematopoietic stem and progenitor cells (HSPC-pDCs), which provides an attainable and large source of pDCs. HSPC-pDCs present pDC characteristics and functions, and like naturally occurring pDCs they exhibit large phenotypic and functional heterogeneity. Here, we characterize different cell subsets from *in vitro* pDC differentiation and identify a distinct population, which is the major producer of IFNα in response to TLR9 stimulation and display a transcriptomic profile similar to what is seen for pDCs circulating in the blood. We also investigate the possibility of rerouting subset specification during HSPCs-to-pDC differentiation by controlling gene expression of key master transcription factors (TFs). We identify TFs associated with the pDC differentiation trajectory that are essential for the development of TLR9-responsive HSPC-pDCs, and we also identify TFs that increase their frequency. In conclusion, we phenotypically and functionally characterize different cell subsets and modulate their relative frequencies by manipulating TF expression during pDC differentiation. These findings provide a deeper understanding of *in vitro*-differentiated pDC cultures that may spur further developments in their use as an immunomodulatory cell therapy.

## Introduction

Plasmacytoid dendritic cells (pDCs) play crucial roles in modulating and controlling both the innate and adaptive immune systems. They are the body’s main producers of type I Interferons (type I IFNs) in response to viral infections, and their functions encompass broad cytokine production, antigen presentation, and cytotoxicity (1–5). The therapeutic potential of pDCs in various cancers is currently under investigation, with clinical trials having been conducted using pDC cell therapy or pDC-targeting agents on patients with metastatic melanoma, prostatic neoplasms, and metastatic endometrial cancer (6–10). Importantly, pDCs can play a dual role in antitumor immunity by both presenting antigens and exerting direct cytotoxic effects (11). Their antitumor immune responses, however, can be impeded by signals from the tumor microenvironment (TME), as has been described in various cancers. These signals, combined with the activation of inhibitory receptors on pDCs, can promote immune tolerance (12, 13). Although their application in cancer immunotherapy is still in the early stages, pDC-based vaccines have already demonstrated a promising safety profile, with minimal toxicity reported. Furthermore, pDC-based vaccines have been proven to induce specific T cell responses against tumor-associated antigens [reviewed in (13)].

Despite the therapeutic potential of naturally blood-circulating pDCs, several obstacles hinder pDC-based cell therapies. These include: (1) the challenge of obtaining sufficient cell numbers from patients due to their low abundance in the blood (pDCs constitute only 0.1-0.5% of human PBMCs), (2) their short lifespan when cultured *ex vivo* (14), and (3) the difficulty in genetically engineering pDCs using for example CRISPR/Cas gene editing. We have previously developed a successful approach for the *in vitro* production of pDCs from CD34+ hematopoietic stem and progenitor cells (HSPCs), providing a readily available source of pDCs (termed HSPC-pDCs) for scientific and therapeutic purposes (14–16). With this *in vitro* differentiation protocol, HSPC-pDCs can be produced in substantial numbers, and gene edited cells can be generated by first modifying CD34+ HSPCs with CRISPR/Cas engineering and then differentiating them into pDCs.

Human pDCs isolated from peripheral blood exhibit large phenotypic and functional heterogeneity. Distinct subsets of pDCs have been identified based on the expression of cell surface markers. For instance, CD2 expression on pDCs from human peripheral blood and bone marrow delineates two functionally distinct subsets (17, 18). Furthermore, in blood pDCs activated with influenza virus, Alculumbre et al. identified three subsets of pDCs based on CD80 and PD-L1 expression, each with functional specialization: P1-pDCs (PD-L1+CD80-) specialized in type I IFN production; P3-pDCs (PD-L1-CD80+) specialized in T cell activation; and P2-pDCs (PD-L1+CD80+), which display an intermediate phenotype (19). Similarly, this functional specialization observed in response to influenza has also been documented in pDCs activated by SARS-CoV-2, which also diversified into P1-, P3-, and P2-pDCs (20).

A sophisticated approach combining droplet microfluidic single-cell assay with scRNA-seq was employed to investigate the type I IFN response in blood pDCs upon activation with the TLR9 agonist CpG-C. In the study, a cluster of pDCs expressing type I IFN, with less than 0.02% IFNα-producing pDCs, emerged shortly after TLR9 stimulation (2 hours post-stimulation). However, in pDCs in the steady state, a specific subset correlating with potential IFNα-producing pDCs was not identified (21). Recently, Ghanem et al. utilized scRNA-seq to explore the transcriptional diversity of blood pDCs, uncovering considerably greater cellular heterogeneity than previously documented. Their analysis identified nine subclusters in non-activated pDCs and up to fifteen unique transcriptional clusters in pDCs activated with influenza virus. Their analysis also showed that a single population of pDCs, comprising 5.5% to 24% of cells, was responsible not only for production of IFNα, but also for other type I and III IFNs (22). In yet another study employing scRNA-seq on blood pDCs isolated from individuals with HIV-1 and healthy controls, researchers initially identified 13 distinct clusters at baseline. Among these, three clusters exhibited expression of genes classically regarded as being specific to pDCs, including CLEC4C, IL3RA, LILRA4, and MZB1. Furthermore, following treatment with a TLR9 agonist, a novel subset of cytotoxic-like pDCs was discerned, characterized by elevated expression of antiviral interferon-stimulated genes (ISGs) and cytotoxic genes compared to the other pDC clusters (23). *In vitro* differentiated pDCs from cord blood CD34+ HSPCs have recently been characterized through scRNA sequencing. The differentiated pDCs exhibited a gene expression profile consistent with the canonical pDC gene expression signature, including TCF4, LILRA4, and CD303. However, a reduction in CD123 expression was observed compared to primary pDCs enriched from PBMCs, which the authors suggest may be due to prolonged IL-3 exposure during culture. Their differentiation protocol resulted in pDCs constituting 23% of the total differentiated cells, which is reflected in the heterogeneity observed within the scRNA-seq data. Transcriptional analysis of the sorted pDC fraction further confirmed its strong similarity to primary pDCs upon TLR stimulation (24).

Consistent with observations in naturally occurring pDCs, cells emerging from *in vitro* HSPC-to-pDC differentiation exhibit heterogeneity based on the expression of the pDC surface markers CD123 and CD303. However, this heterogeneity has to date not been studied in much detail. Here, we characterize three distinct subsets distinguished by varying levels of these markers, suggesting potential differences in their biological functions. We explore the IFN response following TLR9 activation in these subsets and investigate transcriptomic differences with RNA-seq. Using CRISPR/Cas knockout of key master transcription factors (TFs) in HSPC-pDCs, we identify TFs associated with the pDC differentiation trajectory that are essential for the development of TLR9-responsive cells and TFs that increase their frequency. Overall, we phenotypically and functionally characterize distinct cell subsets merging from *in vitro* pDC differentiation, and we demonstrate the feasibility of modulating their relative frequencies by manipulating TF expression during differentiation.

## Results

### An cell subset with high CD123 expression is enriched for IFNα-producing cells in response to TLR9 stimulation

**Figure 1:**
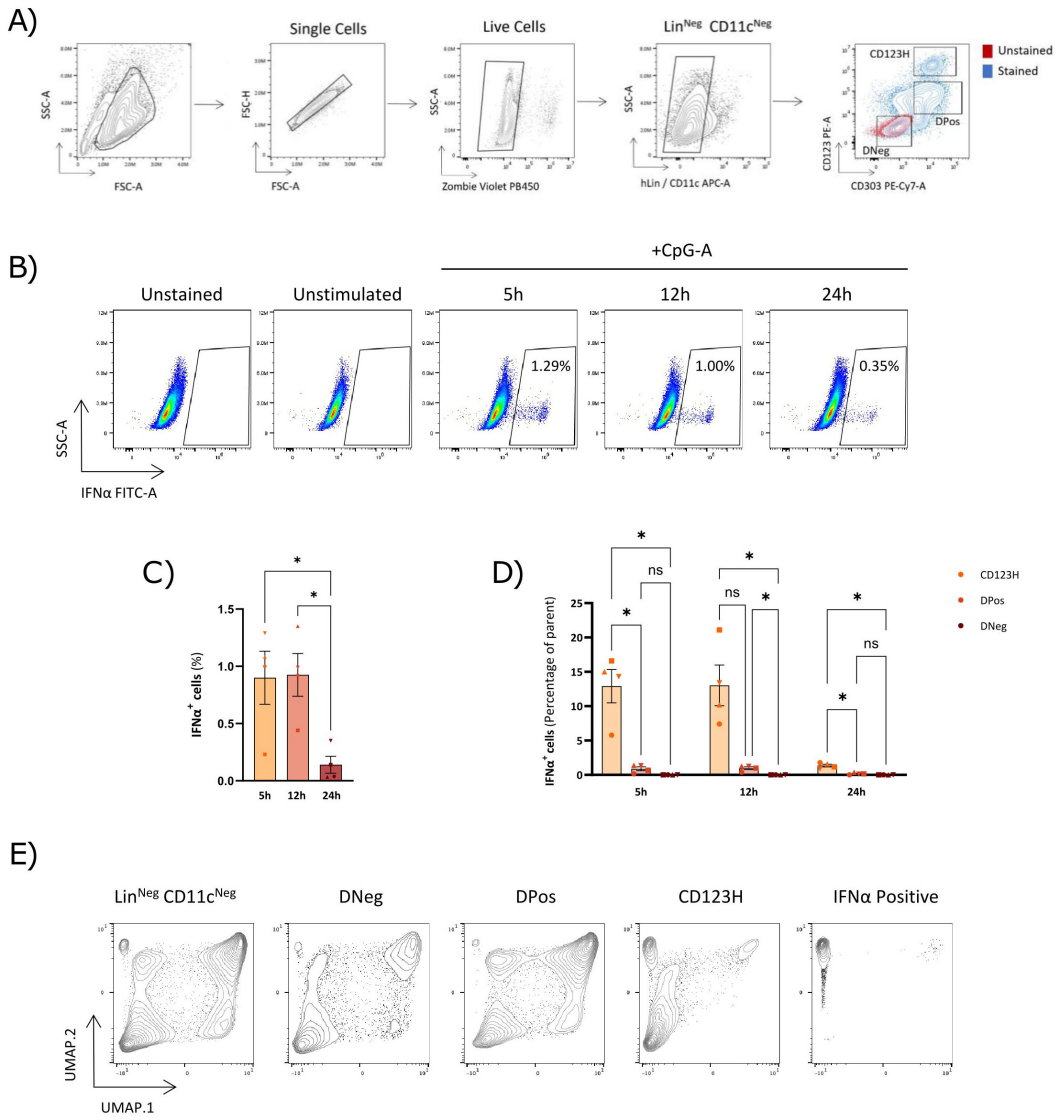
CD123-high HSPC-pDCs are the primary producers of IFNα in response to TLR9 stimulation. **(A)** Gating strategy used to identify HSPC-pDCs. The pDC-related markers CD303 and CD123 are analyzed within viable, lineage-negative, and CD11c-negative cells. **(B)** Representative flow cytometry plots showing IFNα secretion in HSPC-pDCs after TLR9 stimulation. **(C)** Bar graph showing the percentage of IFNα-positive cells within the hLin^-^; CD11c^-^; population at 5, 12, and 24 hours following TLR9 stimulation with CpG-A. **(D)** Bar graph depicting the proportion of IFNα-positive cells within each subset of hLin^-^; CD11c^-^; cells. The data shown represent the mean ± SEM of cells from four donors. Statistically significant differences between groups were determined using Two-Way ANOVA followed with Geisser-Greenhouse correction. *p < 0.05 **(E)** UMAP projections of pDC markers and IFNα gene expression in HSPC-pDCs, using an integrated dataset from HSPC-pDCs derived from multiple donors and stimulated with the TLR9 agonist CpG-A for 5, 12, and 24 hours. ns, non-significant.

We previously used the surface markers CD123 and CD303 to phenotypically characterize cells derived from *in vitro* pDC differentiation cultures. This staining roughly demarcates three subsets: CD123-/CD303- (DNeg), CD123+/CD303+ (DPos), and CD123++/CD303+ (CD123H) (**Figure 1A**). To investigate the expression of IFNα in these subsets and to study the dynamics of IFNα production, we analyzed intracellular IFNα levels by flow cytometry in HSPC-pDCs derived from CD34+ HSPCs from four different donors at different time points after stimulation with the TLR9 agonist CpG-A. Previously, we reported that HSPC-pDCs require priming with IFN-β and IFN-γ to achieve functional activation and responsiveness to TLR7 and TLR9 agonists (14). Accordingly, we primed HSPC-pDCs for 24 hours prior to stimulation with CpG-A. IFNα was measured at 5, 12, and 24 hours after TLR9 stimulation. We observed that around 1% of all cells showed detectable intracellular IFNα, which was detected as soon as 5 hours after stimulation (**Figures 1B, C**). This proportion was maintained at 12 hours post-stimulation but declined markedly at 24 hours. Notably, 5 and 12 hours post-TLR9 stimulation, a mean of 12% of CD123H cells produced IFNα, whereas this was less than 1% of DPos cells (**Figure 1D**). No IFNα production was detected in the DNeg subset. The CD123H subset comprises approximately 5-10% of all HSPC-pDCs depending on donor (Data not shown), and the data show that this subset is enriched for IFNα-producing cells in response to TLR9 stimulation (**Figures 1D, E**).

To further support this observation and confirm that intracellular IFNα was exported from the cells, we sorted the three different HSPC-pDC subsets and analyzed IFNα production in a flow cytometry-based secretion capture assay after priming and 5 hours of stimulation with CpG-A (**Supplementary Figure S1**). Even though this assay can be confounded by background signal from bystander cells that capture IFNα from neighboring producer cells, thereby overestimating the proportion of IFNα-secreting cells, we confirmed high IFNα secretion from the sorted CD123H subset, low secretion from the DPos subset, and no secretion from the DNeg subset (**Supplementary Figure S1c**). These results support the finding that the CD123H subset is enriched for cells with TLR9-responsiveness and IFNα production and secretion capability.

### Transcriptome analysis of HSPC-pDC subsets reveals enhanced TLR9 signaling in CD123H HSPC-pDCs

**Figure 2:**
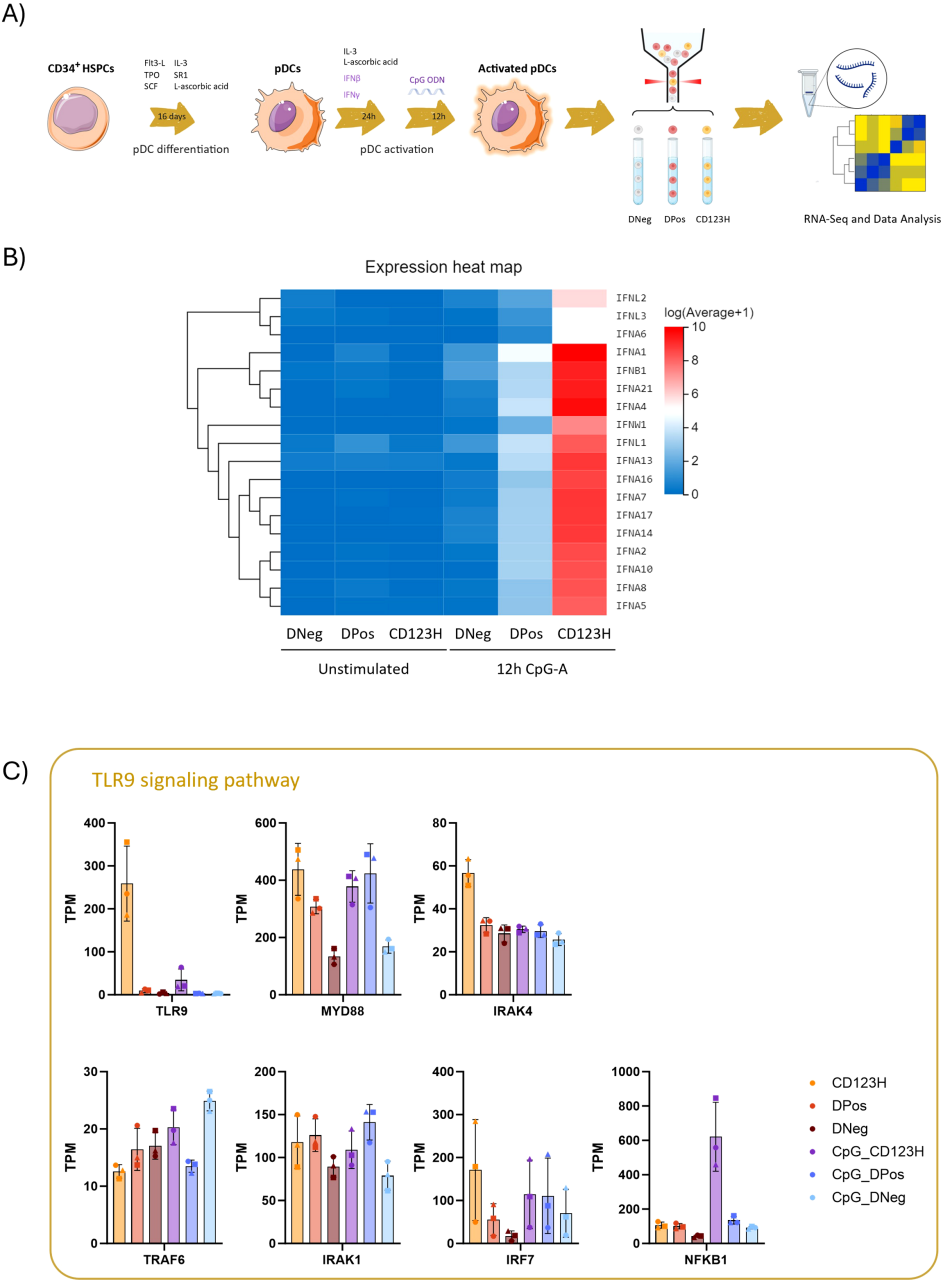
Characterization of cell subsets derived from *in vitro* pDC differentiation by RNA-seq **(A)** Schematic diagram illustrating the experimental design for this study. Following 16 days of HSPC-to-pDC differentiation, HSPC-pDCs obtained from three donors were primed with IFN-β and IFN-γ for 24 hours and then stimulated with the TLR9 agonist CpG-A for 12 hours. A primed but unstimulated condition was included for each donor. Based on the expression of CD303 and CD123, the three subsets (DNeg, DPos, and CD123H) were sorted from unstimulated and stimulated cells. RNA was extracted from the sorted samples for RNA-seq analysis **(B)** Heat map illustrating RNA-Seq expression data for Type I and Type III IFNs within the three sorted subsets. **(C)** Bar graph displaying Transcripts Per Million (TPM) counts for various genes involved in the TLR9-mediated immune signaling pathway. Upon ligand binding, TLR9 initiates downstream signaling via MyD88, leading to the activation of IRAK4. Subsequently, IRAK4 recruits IRAK1 and TRAF6, which further promotes the type I IFN, NF-κB, and MAPK signaling pathways.

Next, by combining cell sorting with RNA sequencing (RNA-seq), we aimed to unravel the differences in the expression profiles between the three cellular subsets. For this purpose, we sorted the subsets from three donors following priming, with or without 12 hours of TLR9 stimulation with CpG-A (**Figure 2A**). Following RNA sequencing, we analyzed the transcriptome profile and differentially expressed genes (DEGs) of each subset (the 50 most significant DEGs between groups are presented as heatmaps in **Supplementary Document S1** in **Supplementary Data Sheet 1** and **Supplementary Table 2** lists all DEGs between groups). The three subsets did not appear to align with the pDC subsets previously proposed based on PD-L1 and CD80 expression. Additionally, the marker CD2, which has been linked to pDC heterogeneity, showed relatively low expression levels across the three subsets and did not distinguish them (data not shown).

Consistent with our prior findings on IFNα protein production, when examining the transcripts of all 17 subtypes of type I and type III IFNs, the CD123H subset exhibited the highest production of IFNs whereas intermediate expression was observed in the DPos subset and none in the DNeg population (**Figure 2B**). This observation was validated by RT-qPCR with primers specific for both IFNA-1 and -13 in subsets sorted after 4 or 20 hours after TLR9 stimulation with CpG-A (**Supplementary Figure S2**).

Analysis of gene transcripts associated with the TLR9 signaling pathway revealed high expression of TLR9 in the CD123H subset, contrasting with very low levels in the DPos and DNeg subsets (**Figure 2C**, **Supplementary Figure S3**). GSEA analysis showed that CD123H cells have an enrichment score of 0.42 for the TLR signaling pathway after CpG-A stimulation, compared to the DPos subset, reflecting an enrichment of genes related to this pathway in CD123H cells (**Supplementary Figure S3**). Additionally, the TLR9 downstream adaptor protein MyD88 as well as IRF7, which mediates production of type I IFN via MyD88, showed higher expression levels in unstimulated CD123H HSPC-pDCs compared to DPos and DNeg HSPC-pDCs (**Figure 2C**). NF-kappa-B (NFKB1), which upon activation by MyD88 and TRAF6 induces the expression of proinflammatory cytokines and upregulates costimulatory molecules, also showed higher expression in CD123H HSPC-pDCs following TLR9 stimulation compared to DPos and DNeg subsets (**Figure 2C**).

When analyzing cytokine and chemokine gene expression in CpG-A-stimulated *versus* non-stimulated cells, all three cell subsets were found to respond to TLR9 stimulation (**Supplementary Figure S4A**). Although the profiles of upregulated cytokines and chemokines differed among the subsets, some proinflammatory cytokines and chemokines, such as IL6, IL36G, CCL8, CXCL11, and CCL2 were evident across all three subsets (**Supplementary Figure S4A**). However, as CXCL11 and CCL8 are ISGs, these may not be induced in direct response to CpG-A. The upregulation of canonical ISGs was also observed following TLR9 stimulation across all three subsets, relative to non-stimulated HSPC-pDCs (**Supplementary Figure S4B**). Reactome pathway analysis of TLR9-activated cells indicated a significant upregulation of IFN signaling and related pathways in CD123H HSPC-pDCs compared to both DNeg and DPos subsets. Moreover, CD123H HSPC-pDCs appeared to downregulate genes involved in the cell cycle (**Supplementary Figure S4C**). When comparing DPos to DNeg subsets, we found that innate immune system pathways and TLR cascades, among other pathways, were upregulated in DPos cells (**Supplementary Figure S4C**).

### Characterization of transcription factor expression in HSPC-pDC subsets

**Figure 3:**
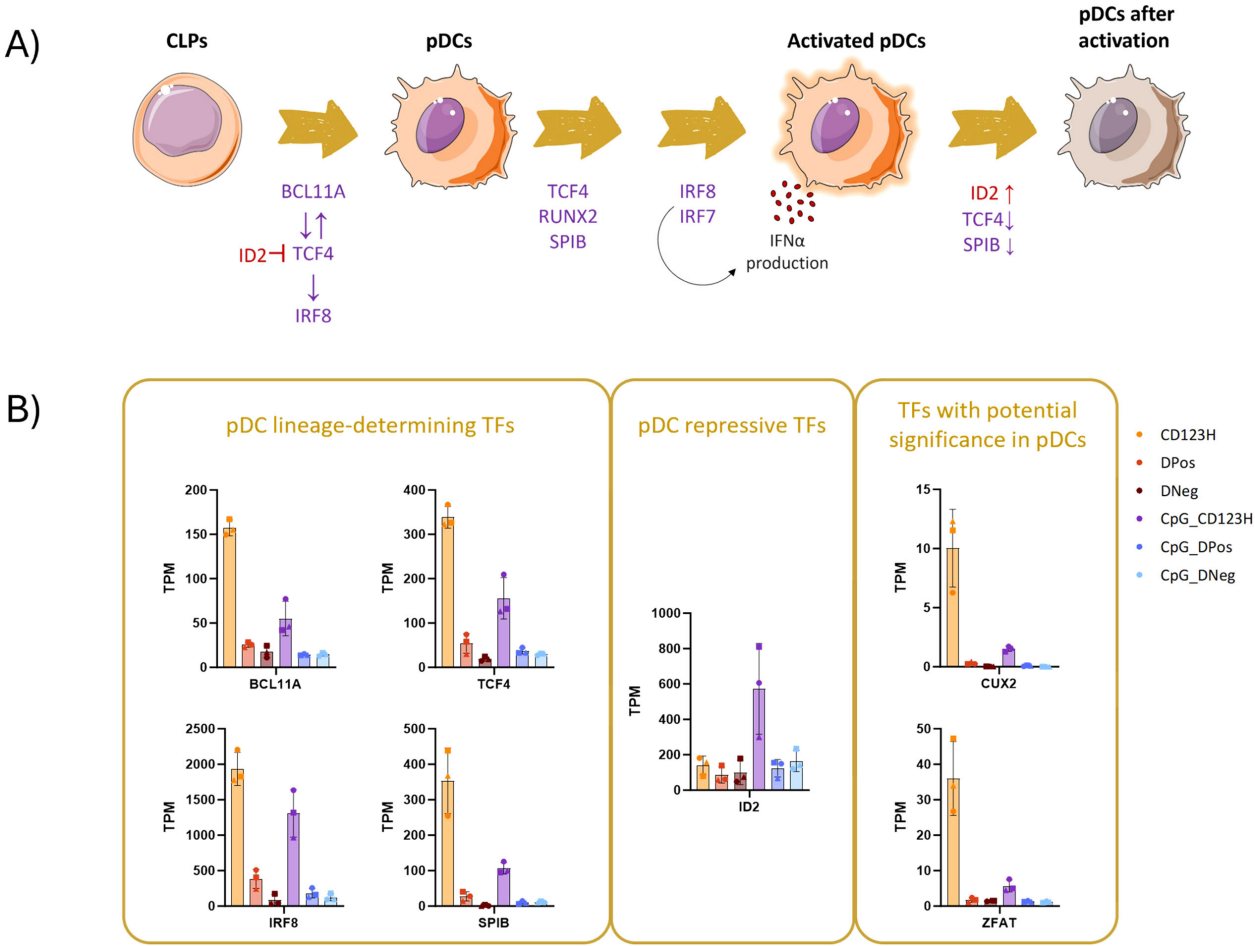
Unraveling the transcription factor network in development of HSPC-pDCs. **(A)** Diagram depicting the network of transcription factors that regulate the development and function of blood pDCs. **(B)** Bar graph displaying Transcripts Per Million (TPM) counts of different transcription factors associated with pDCs: BCL11A, IRF8, SPIB, and TCF4 (left square); the pDC-repressive transcription factor ID2 (middle square); and two transcription factors identified as differentially expressed genes (DEGs) in the CD123H subset: CUX2 and ZFAT (right square).

Various transcription factors (TFs) regulate the development, maturation, and cellular function of pDCs. Among these, TCF4 and SPIB have been reported to be required for pDC differentiation, and in conjunction with IRF7 and IRF8, they mediate the IFN response following TLR stimulation (**Figure 3A**). We therefore used the RNA-seq data to profile the expression of TFs across the three subsets to elucidate the differences underlying their heterogeneity. Our analysis revealed differential expression of key TFs previously associated with pDC development and function in the CD123H subset, both in unstimulated and TLR9-stimulated HSPC-pDCs. The transcription factors BCL11A, IRF8, TCF4, and SPIB, which are traditionally associated with the specification and function of pDCs, exhibited higher expression in the CD123H subset compared to the DPos subset in unstimulated cells (**Figure 3B**). The pDC-suppressive TF ID2 was expressed at equal levels among the three subsets before TLR9 stimulation but increased in the CD123H subset following stimulation (**Figure 3B**). This upregulation of ID2 and downregulation of pDC-specifying TFs following TLR9 stimulation has been documented in naturally occurring pDCs (25, 26). RUNX2, another TF essential for the expression of genes characteristic for pDCs (27, 28), was also upregulated in CD123H cells compared to the DPos and DNeg subsets (**Supplementary Document S1** in **Supplementary Data Sheet 1**).

Additionally, among the 50 most significant DEGs between the CD123H and DPos subsets, we identified the TFs CUX2 and ZFAT (**Figure 3B**, and **Supplementary Document S1** in **Supplementary Data Sheet 1**). These TFs have previously been associated with pDCs, although their specific functions remain unclear to date (29, 30). However, in all subsets, CUX2 was expressed at relatively low levels, but such findings suggest that both TFs may play roles in pDC biology.

### Cell type enrichment scores using RNA deconvolution show substantial enrichment of progenitor-like cells

**Figure 4:**
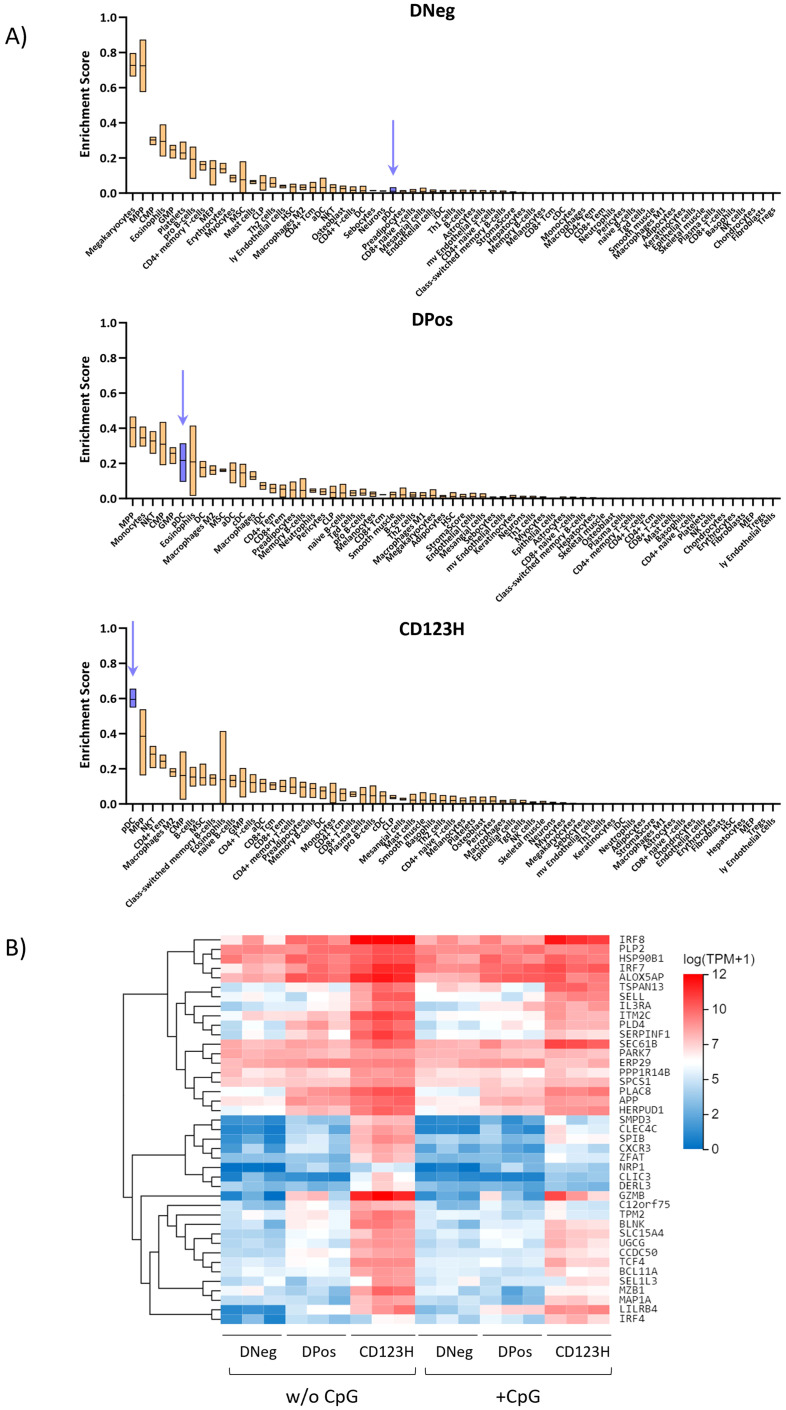
Cell type enrichment scores using RNA deconvolution of subsets derived from *in vitro* pDC differentiation. **(A)** Boxplots showing the enrichment scores for different cell types across each subset after 16 days of differentiation (DNeg, DPos, and CD123H) as predicted by the xCell RNA Deconvolution Method. This computational approach scores cell type resemblances from bulk transcriptomic data. **(B)** Heat map showing RNA-Seq expression data for genes associated with the expression profile of blood pDCs within the three subsets.

To give further clues to the identity of the different cell populations, we used xCell to conduct an RNA deconvolution analysis with our RNA-seq data from the three subpopulations. The xCell tool uses cell type-specific expression signatures from multiple data sources to score the resemblance of a transcriptome profile to different cell types from 0 to 100% (31). This analysis showed that out of the 65 included reference cell types, the DNeg subset had the highest resemblance score to megakaryocytes/platelets, multipotent progenitors (MPPs), common myeloid progenitors (CMP), eosinophils, and granulocyte/monocyte progenitors (GMPs) (**Figure 4A**). Interestingly, the resemblance score to blood pDCs was close to 0%. The DPos subset had some of the same resemblance hits as the DNeg subset, but interestingly the resemblance score to blood pDCs increased to 22% indicating a much higher pDC-like transcriptome. The CD123H subset showed highest resemblance to blood pDCs with a resemblance score of 60%. Overall, these analyses suggest that the DNeg population mostly shares transcriptomic characteristics with progenitor cells, the DPos subset shares transcriptomic characteristics with both progenitor cells and blood pDCs, and the CD123H subset mainly have blood pDC transcriptome characteristics. Nevertheless, RNA deconvolution provides only a partial portrayal of cellular heterogeneity and should be interpreted with caution as its resolution falls short of the precision achievable with techniques like single-cell RNA sequencing. To support these results, gene expression analysis of genes previously associated with the blood pDC expression profile (32) across the three subsets revealed that the CD123H cells displayed the highest overall expression of these genes (**Figure 4B**).

**Figure 5:**
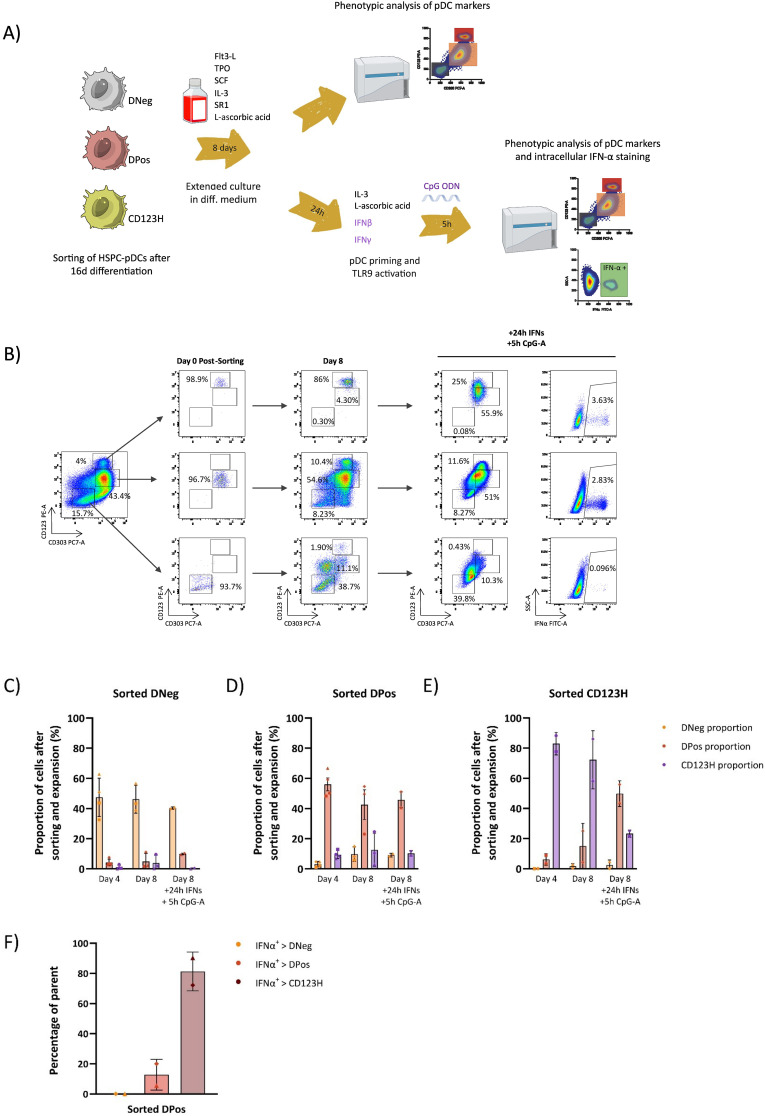
Differentiation potential of subsets obtained during HSPC-to-pDC differentiation. **(A)** Schematic representation of the experimental design: After 16 days of differentiation, unstimulated cells were sorted into DNeg, DPos, and CD123H subsets based on CD303 and CD123 antibody staining. These subsets were then cultured in differentiation medium for up to eight additional days followed by priming and TLR9 stimulation. Their phenotype and TLR9 responsiveness were assessed through cell surface staining and intracellular IFN-α staining. **(B)** Flow cytometry plots showing CD123 and CD303 expression in bulk cells prior to sorting (left), and in sorted cells immediately post-sorting, after 8 additional days of differentiation, and following TLR9 stimulation (middle panels). The right panels show IFN-α expression in sorted cells after 8 additional days of culture and subsequent TLR9 stimulation. **(C-E)** Bar graphs showing the frequencies of DNeg, DPos, and CD123H subsets within sorted subsets after an additional four and eight days of culture in differentiation media and subsequent TLR9 stimulation. Each symbol corresponds to a different donor. Phenotypic analysis was performed at both time points after sorting for some donors, whereas for others, analysis was restricted to only one time point due to the limited number of available cells. Data are presented as mean ± SEM. **(F)** Bar graph showing the proportion of IFN-α-positive cells within each fraction of DNeg, DPos, and CD123H cells that emerged from the sorted DPos subset after 8 days of further culturing, priming, and CpG-A stimulation. For example, the bar with the darkest shade (IFNa^+^ > CD123H) means that 81% of all IFN^+^ cells were located in the CD123H subpopulation that had emerged from the sorted DPos population. IFNα intracellular staining was performed 5 hours after stimulation with CpG-A. Data are presented as mean ± SD.

These findings could suggest that the CD123H pDC subset represents a type of mature pDC, while the DNeg and DPos subsets may correspond to progenitor cells and pDC precursors at an earlier maturation stage, respectively. To test this hypothesis, we performed a new sorting of the three subsets after 16 days of differentiation. The sorted cells were then maintained in differentiation medium for up to eight additional days, after which their phenotype was analyzed (**Figure 5**, **Supplementary Figure S5**). After this period, only around 45% of the sorted DNeg fraction preserved its phenotype while most of the remaining cells had gained intermediate CD123 expression levels, while maintaining low levels of CD303 (**Figures 5B, C**). A small fraction of the cells had transitioned to the DPos or CD123H phenotype. In contrast, up to 20% of the sorted DPos subset transitioned to CD123H, whereas the majority of CD123H cells retained their phenotype (**Figures 5D, E**). We also tested the capacity of these sorted and 8-day expanded cells to produce IFNα following TLR9 stimulation with CpG-A. Notably, the DNeg fraction still showed a very limited capacity to produce IFN-α (<0.1%), whereas the DPos fraction demonstrated IFNα production comparable to or even surpassing that of CD123H HSPC-pDCs (**Figure 5B**, **Supplementary Figure S5**). The IFN-α-producing cells within the sorted and further cultured DPos subset were mainly found in the newly emerged CD123H fraction (**Figure 5F**). Despite low cell sorting yields limiting data collection for some conditions and time points, the overall findings provide evidence that DPos cells have the potential to transition into CD123H HSPC-pDCs. These newly formed CD123H HSPC-pDCs demonstrate the capacity to produce IFN-α following TLR9 stimulation. In contrast, there was no significant change in the phenotype of sorted CD123H HSPC-pDCs after an additional 8 days of culture. However, CD123 expression levels decreased in sorted CD123H HSPC-pDCs following TLR9 stimulation, with minimal IFN-α production observed in one donor (**Supplementary Figure S5**). These findings suggest that, rather than representing discrete populations, the three subsets exhibit some degree of phenotypic plasticity, which may allow for transitions between subsets in response to varying conditions.

### pDC-related transcription factors are critical for type I interferon production in HSPC-pDCs

Previous studies have shown that the loss of TFs known to be directly involved in pDC development results in impaired pDC generation. Conversely, upregulation of those TFs has the opposite effect (33). Therefore, we reasoned that one way to assess the role of relevant TFs in HSPC-pDCs identity was to use CRISPR/Cas to knock out relevant candidate TFs and examine the phenotype and TLR9-responsiveness of the resulting cells.

**Figure 6:**
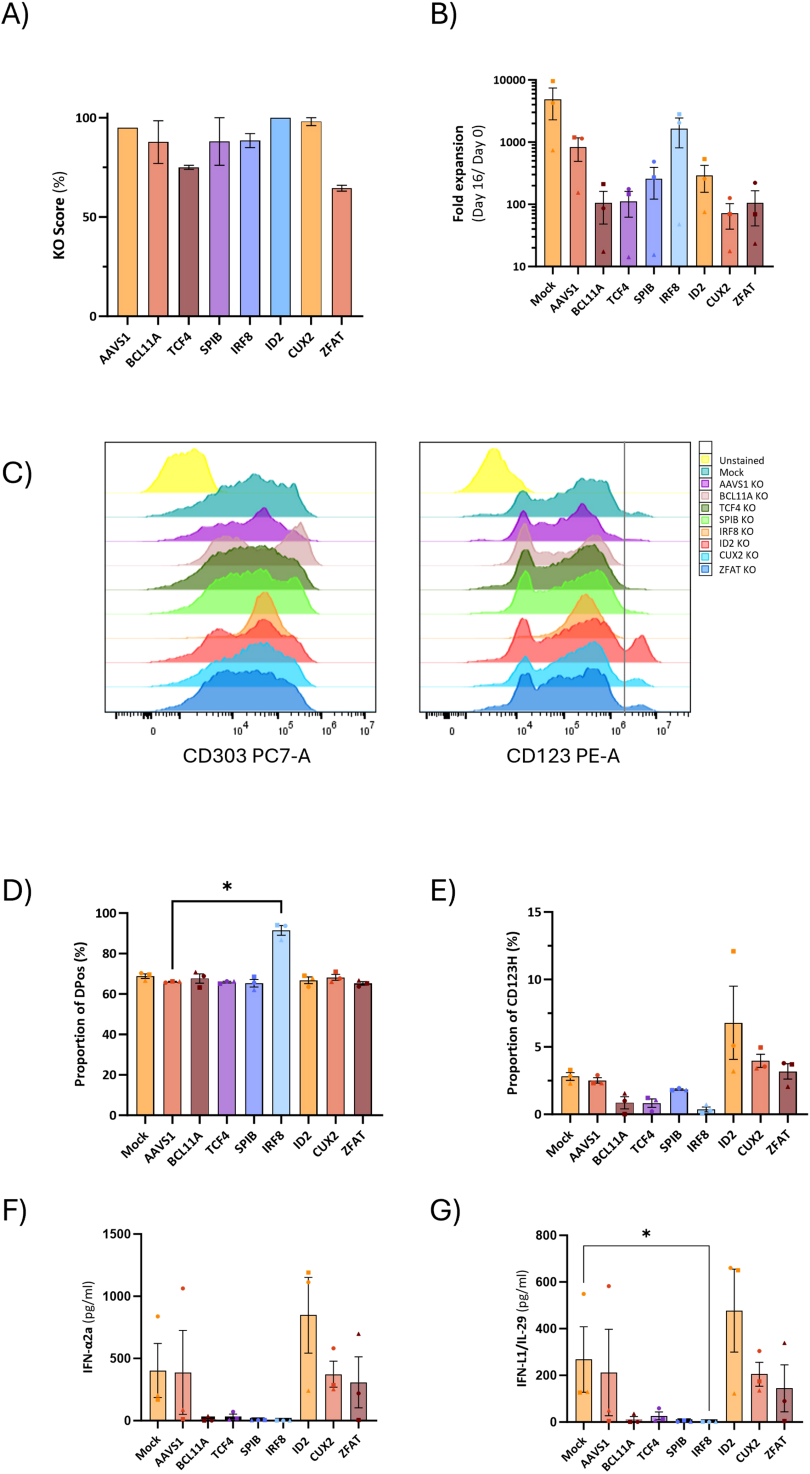
Yield and phenotypic impact of knockout of TFs conventionally associated with pDCs and TFs with potential significance for pDCs during HSPC-to-pDC differentiation. **(A)** Knockout efficiencies in HSPC-pDCs for each TF associated with pDCs. Genomic DNA samples were obtained 3-5 days after nucleofection, and indel frequencies were determined by ICE analysis. **(B)** Graph illustrating the fold expansion of HSPC-pDCs cultured over a 16-day differentiation period. Data points represent mean values ± SEM of three donors. **(C)** Representative flow cytometry histograms illustrating the surface expression of CD303 and CD123 on knockout HSPC-pDCs. HSPC-pDCs were previously primed with IFN-β and IFN-γ for 24 hours. **(D, E)** Bar graphs illustrating the percentages of DPos and CD123H cells following priming with IFN-β and IFN-γ for 24 hours. Data shown represent mean ± SEM of three donors. One-way ANOVA was used to analyze differences between groups. **(F, G)** Levels of IFN-α2a and IFN-λ1/IL-29 in gene-edited HSPC-pDCs after stimulation with CpG-A. Data shown represent the mean ± SEM of a minimum of three donors. Differences between groups were analyzed using the Kruskal-Wallis test. * p < 0.05.

**Figure 7:**
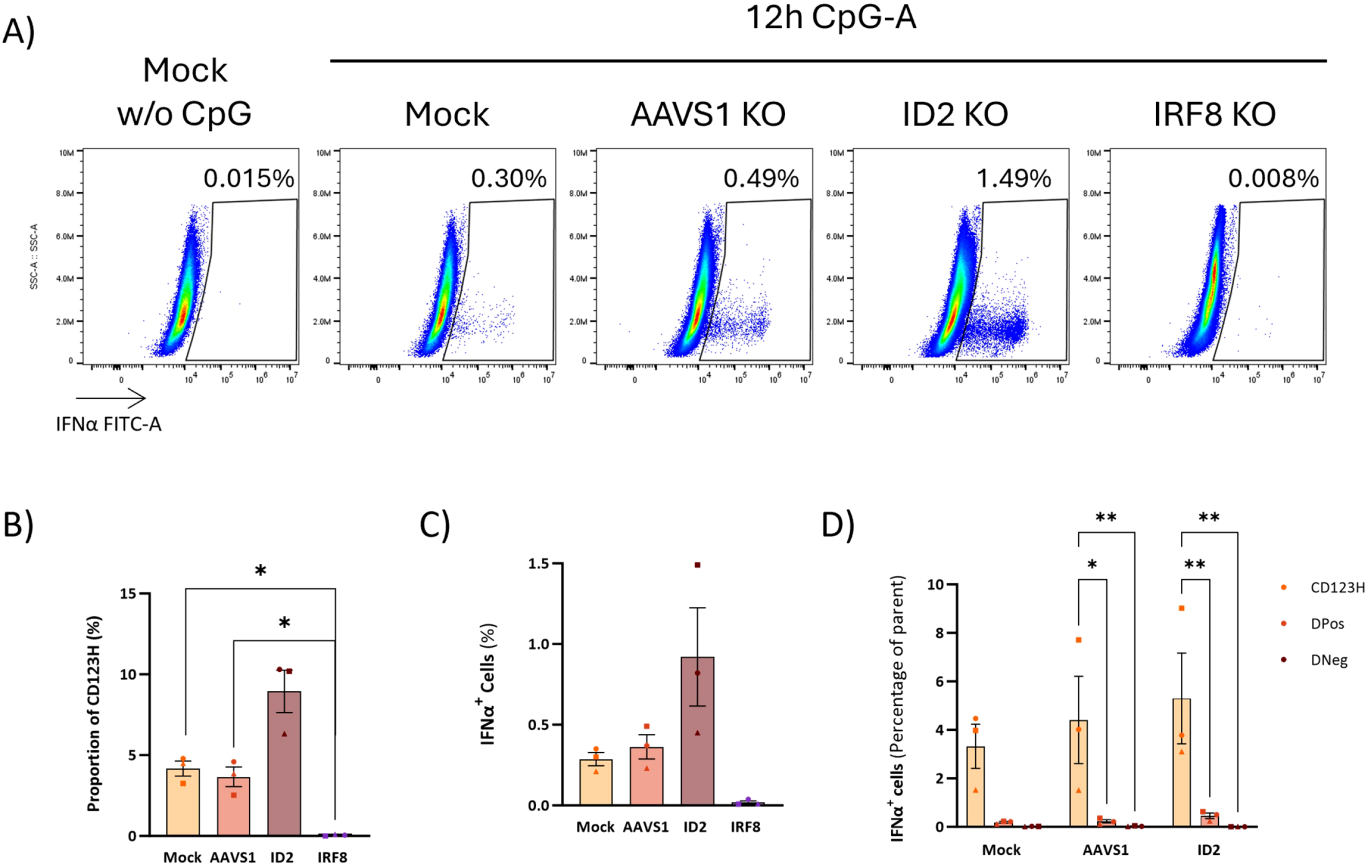
Role of pDC-related TFs in IFNα production upon TLR9 activation. **(A)** Representative flow cytometry plots showing IFNα production in ID2 KO and IRF8 KO HSPC-pDCs. Mock and AAVS1 KO HSPC-pDCs were included as controls. IFNα intracellular staining was performed 12 hours after stimulation with CpG-A. **(B)** Graph showing the percentage of CD123H HSPC-pDCs 12 hours after stimulation with the TLR9 agonist CpG-A. **(C)** Graph illustrating the percentage of IFNα-producing cells. Data shown represent mean ± SEM of three donors. One-way ANOVA was used to analyze differences between groups. **(D)** Graph illustrating the proportion of IFNα-positive cells within each cell subset derived from *in vitro* pDC differentiation. Statistically significant differences between groups were determined using Two-Way ANOVA. *p < 0.05, **p < 0.01.

In our previous work, we demonstrated that it is possible to obtain genetically modified pDCs derived from CRISPR/Cas9 gene-edited CD34+ HSPCs (14). Based on the RNA sequencing data, we selected four pDC-promoting TFs (BCL11A, TCF4, SPIB, and IRF8), the pDC-repressing transcription factor ID2, which inhibits TCF4, and the two TFs with unknown significance in pDC biology (CUX2 and ZFAT). We designed CRISPR/Cas sgRNAs targeting these 7 genes and introduced them together with Cas9 protein into CD34+ HSPCs and evaluated gene knockout (KO) by Sanger sequencing of the targeted genomic loci followed by ICE analysis to quantify the percentage of alleles with KO-generating indels. All 7 genes showed KO frequencies of >60% (**Figure 6A**). As a negative control, a sgRNA targeting the safe harbor locus AAVS1 was included. Cells were then differentiated into HSPC-pDCs while cell numbers were measured to assess the extent of expansion during differentiation. These data showed that the expansion of cells was negatively affected under all conditions compared to the Mock group, including the control where the AAVS1 locus was edited. Although these differences were not statistically significant, this trend was observed under all conditions, potentially more pronounced with the loss of BCL11A, TCF4, and CUX2 (showing a 7.8, 7.4, and 11.6-fold reduction, respectively, when compared to the AAVS1 control) (**Figure 6B**). Phenotype evaluation by flow cytometry at the end of differentiation showed that KO of the master regulators BCL11A, TCF4, SPIB, and IRF8 led to a reduction or almost complete loss of the CD123H fraction (**Figures 6C–E**, **Supplementary Figure S6**). In contrast, ID2 KO resulted in an approximately 2.4-fold increase in the proportion of cells within the CD123H fraction compared to controls (**Figure 6E**, **Supplementary Figure S6**). To investigate the effect of TF KO on TLR9 responsiveness, we quantified IFN-α2a and IFN-L1 protein production by Meso Scale multiplex assay following TLR9 stimulation. These data showed that cells with knockout of BCL11A, TCF4, SPIB, or IRF8 lost their ability to produce IFN-α2a and IFN-L1 following TLR9 stimulation. In contrast, ID2 KO HSPC-pDCs exhibited a two-fold increase in IFN-α2a and IFN-L1 production (**Figures 6F–G**). For CUX2 and ZFAT KO, no differences were observed relative to controls. To validate the finding that ID2 knockout increased the CD123H subset with a consequent increase in IFNα production following TLR9 stimulation, we generated three new donors with ID2 knockout and included the IRF8 knockout as a control condition for loss of the CD123H subset and TLR9 responsiveness. Consistent with our previous results, ID2 knockout increased the proportion of CD123H cells by ∼2-fold compared to Mock and AAVS1 controls, however without statistical significance, whereas IRF8 knockout abolished generation of CD123H cells (**Figure 7B**). IFNα levels were measured by intracellular flow cytometry 12 hours after TLR9 stimulation, and consistent with our previous observations using MesoScale multiplex assay and the observed increase in the proportion of CD123H cells, ID2 knockout seemed to increase the overall proportion of cells expressing IFNα but again without reaching statistical significance (**Figures 7A–C**). In contrast, IRF8 knockout did not lead to any IFNα-producing cells following TLR9 stimulation (**Figures 7A-C**). Further analysis of the IFNα-producing cells showed that this increase was not accompanied by a statistically significant increase in the percentage of cells expressing IFNα within each subset of ID2 KO cells. Still, the proportion of CD123H cells expressing IFNα was significantly higher than the percentage of IFNα-producing DPos and DNeg cells disregarding the condition (**Figure 7D**).

### Flow cytometric detection of CD303 on the cell surface is significantly affected by the blocking procedure

**Figure 8:**
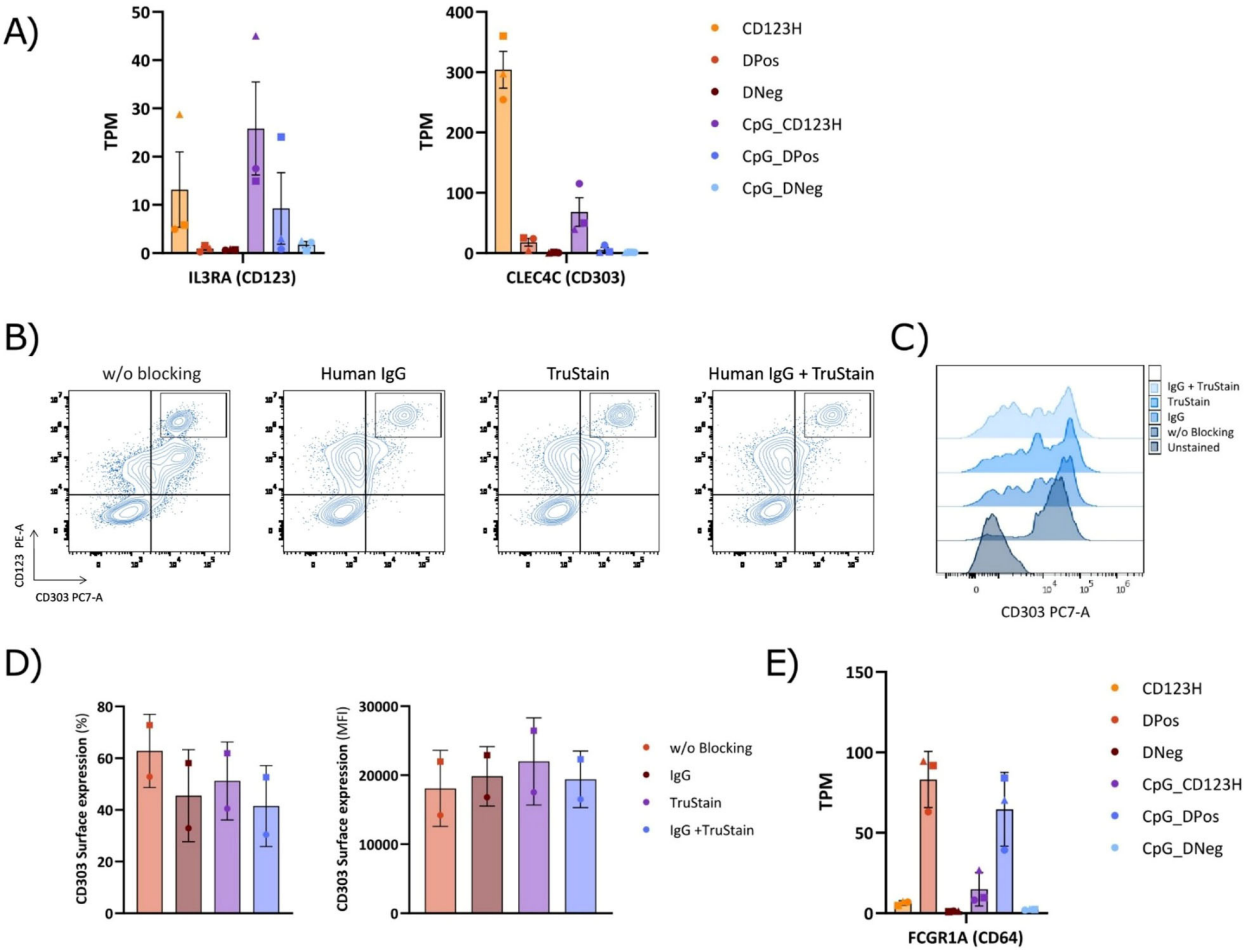
Comparison of Fc receptor blocking methods in the cell surface staining of subsets derived from HSPC-to-pDC differentiation. **(A)** Bar graph displaying Transcripts Per Million (TPM) counts of the human pDC markers CD123 (IL3RA) and CD303 (CLEC4C) in each subset. **(B)** Representative flow cytometry plots showing the cell surface expression of CD123 and CD303 in cells on day 16 of differentiation, employing human IgG alone, TruStain alone, or a combined blocking approach. **(C)** Flow cytometry histograms illustrating the CD303 expression profile obtained following the indicated blocking method. **(D)** Bar graphs showing the proportion of CD303 positive cells within hLin and CD11c negative cells (left) and the CD303 surface expression levels (MFI) (right) using various blocking methods during surface staining. Data are presented as mean ± SD from two donors. **(E)** Bar graph showing Transcripts Per Million (TPM) counts of the Fc gamma receptor I (Fc γRI; FCGR1A).

Analyzing CD123 and CD303 surface receptor expression at the RNA level from the RNA-seq data confirmed that CD123 transcripts correlated with the measured surface protein observed by flow cytometry (**Figure 8A**). However, expression of CD303 mRNA transcripts was surprisingly low in the DPos subset, and almost at the same level as in the DNeg subset, contrasting with the phenotype observed by flow cytometry. We hypothesized that this discrepancy could result from non-specific staining by the anti-CD303 PE-Cy7 antibody, possibly due to Fc receptors (FcRs) binding the Fc region of the antibody or the cyanine dye (34, 35). Blocking agents used to prevent such non-specific antibody binding can be crucial to achieve reliable antibody staining, but our previous efforts using conventional FcR blocker for HSPC-pDC phenotyping showed no difference in staining (data not shown). However, we decided to test two other FcR blocking reagents, Human IgG and Human TruStain FcX reagent, to determine if CD303 detection was confounded by unspecific antibody binding. Both reagents reduced signal from the anti-CD303 PE-Cy7 antibody within the DPos subset, and when combining the two, CD303 staining almost disappeared, without affecting CD303 staining of the CD123H population (**Figures 8B–D**). The CD303 staining of HSPC-pDCs was further assessed using the same anti-CD303 antibody clone (clone 201A) conjugated to BV421 but from a different vendor, and a different anti-CD303 antibody (REAfinity, PE-Vio770, clone REA693) specifically developed to eliminate the need for FcR blocking (**Supplementary Figure S7**). In addition to Human IgG, we here tested the True-Stain Monocyte Blocker developed to prevent non-specific interaction between Cyanine tandem dyes and monocytes and macrophages. Consistent with the results obtained using the PE-Cy7 antibody, both new antibodies exhibited high levels of CD303 staining without blocking. However, the REAfinity antibody required both blocking reagents to significantly reduce CD303 staining whereas the 201A clone conjugated to BV421 only required one blocking reagent. To corroborate this finding, we finally analyzed the expression of FcRs from the RNA-seq data set, which showed >10-fold higher expression of the high-affinity FcγRI (CD64) in the DPos subset compared to the other two subsets (**Figure 8E**). These observations reveal that the cell population previously identified as DPos in the absence of an appropriate FcR blocker, in fact corresponds predominantly to a CD303Neg population with intermediate CD123 levels.

## Discussion

HSPC-pDCs hold significant promise for clinical applications, particularly in immunotherapy for cancer, infectious diseases, and autoimmune disorders due to their unique ability to produce type I IFNs and modulate immune responses. These cells could be harnessed to enhance antiviral immunity, improve vaccine efficacy, or induce immune tolerance in autoimmune diseases. However, translating this technology into clinical practice faces several challenges, including ensuring the scalability and reproducibility of HSPC-pDC production, maintaining their functional stability and safety *in vivo*, and overcoming potential immunogenicity or off-target effects. Long *in vitro* differentiation cultures, regulatory hurdles, and the need for robust preclinical models to demonstrate efficacy and safety could further complicate the path to clinical translation. Despite these hurdles, forthcoming efforts may pave the way for the successful use of HSPC-pDCs in immunotherapeutic applications.

Heterogeneity of human pDCs has previously been documented, revealing distinct transcriptional profiles and functions at single cell resolution (21–23, 32, 36). In the current study, we have phenotypically and functionally characterized three distinct subsets of cells from *in vitro* pDC differentiation of HSPCs. Focusing on the surface markers CD123 and CD303, we discovered a subset of cells with high CD123 expression, that shows the highest resemblance to pDCs that naturally circulate the blood and display the highest degree of TLR9 responsiveness. Whether the other subsets would reassemble tissue-resident or bone marrow pDCs or precursors is currently an unresolved question. At the transcriptional level, we observed an enrichment of several genes traditionally associated with pDCs in the CD123H fraction, including genes associated with the type I IFN response pathway (e.g. TLR9, TLR7, IRF7, and PACSIN1), LILRA4, LAMP5, and granzyme B (GZMB). Indeed, expression levels of GZMB were notably elevated in unstimulated CD123H cells, but it is well established that human pDCs are an abundant source of GZMB in response to IL-3 (22, 37). The fact that only a fraction of HSPC-pDCs elicits the production of type I IFN upon TLR9 stimulation aligns with previous studies showing that only a small proportion of activated human blood pDCs produces type I IFN (21, 22, 38). Among the 13 subtypes of human IFNα, the upregulation of IFNA6 after 12 hours of activation was notably less prominent compared to the other subtypes. It has been observed that IFN6 exhibits a stronger antiviral activity; however, its expression is notably diminished in individuals infected with HIV (39). This phenomenon could elucidate why IFN6 is expressed to a lesser extent in HSPC-pDCs.

In human pDCs isolated from PBMCs, it has been observed that only a small fraction (≈ 1-3%) of pDCs act as key drivers of the IFN response upon TLR activation, a subset referred to as first responders (38). Our findings might suggest that HSPC-pDCs exhibiting first responder characteristics after TLR9 stimulation are predominantly located within the CD123H subset. However, further studies implementing higher time resolution and single-cell analysis would be needed for conclusive evidence of this. Conversely, cells expressing intermediate levels of CD123, initially identified as DPos, display a much lower response to TLR9 activation, but when isolated and cultured further, they could transition into the CD123H phenotype and gain higher TLR9 responsiveness with secretion of type I IFN. In addition, some TLR9-responsive cells were present in this population. It is well established that CD123 is expressed on progenitor cells in human blood (40, 41). In accordance, our observations suggest the presence of both early-stage pDC precursors within this subset and the presence of a small TLR9-responsive subset of pDCs. These two populations might overlap, which could be the focus of future studies. In the deconvolution analysis, the CD123H subset exhibited a transcriptomic profile closely aligned with that of blood pDCs. Nonetheless, transcriptome comparisons between freshly isolated cells and *in vitro* cultured cells are challenged by the large differences in extrinsic conditions such as nutrients, cytokines, gases (O_2_, N_2_, and CO_2_), and pH that might impact our maturation protocol so that is does not fully replicate physiological signals.

The commitment and development of pDCs are regulated by various TFs, including BCL11A, TCF4, SPIB, and IRF8. Our RNA-seq analysis of sorted cell subsets derived from *in vitro* pDC differentiation revealed increased expression levels of these TFs within the CD123H subset compared to the other subpopulations. Notably, the expression levels of these pDC-related TFs were downregulated following CpG-induced activation of HSPC-pDCs, while the expression of ID2, which antagonizes TCF4, a master regulator of pDCs, increased. The downregulation of genes associated with the pDC lineage, along with the adoption of transcriptomic characteristics typical of cDCs, has previously been documented in activated blood pDCs (22, 42–44).

One major advantage of our *ex vivo* setup for generating HSPC-pDCs lies in the ease with which these cells can be genetically modified in contrast to blood pDCs (14, 16). Although nucleofection of human pDCs from peripheral blood mononuclear cells (PBMCs) with CRISPR-Cas9 ribonucleoprotein is feasible, it highly impacts the recovery of the pDCs (45), which could potentially affect their functionality. In contrast, our setup allows us to utilize CRISPR/Cas to delve into the functional roles of specific genes in HSPC-pDCs without significantly impacting pDC fitness or biology. The only negative effect observed from CRISPR/Cas gene editing was a reduction in the number of HSPC-pDCs generated, observed with both the knockout of different TFs and in the AAVS1 control which we believe can be ascribed to the impact of electroporation on the initial expansion of progenitor cells. To evaluate the importance of pDC-related TFs we generated various knockout HSPC-pDCs using CRISPR/Cas9 in multiple donors. The knockdown of BCL11A, TCF4, SPIB, and IRF8 in HSPCs led to a substantial decrease or almost complete depletion of the CD123H fraction. Moreover, the loss of these TFs also resulted in the inability to produce type I IFNs after TLR9 stimulation. The critical role of these TFs in the development and TLR9-related functionality of pDCs is well-documented, and previous studies have shown that the loss of these TFs leads to a reduction in pDC numbers and type I IFN production, both *in vitro* and *in vivo* (25, 33, 46). Prominent evidence is the impaired development of pDCs observed in individuals with Pitt-Hopkins syndrome, caused by TCF4 haploinsufficiency (25). To investigate the role of specific TFs in the development of human pDCs, retroviral vectors have been used in CD34+ cells to introduce either the expression of TF cDNA or repress them using RNAi (33, 46). This approach allows for the overexpression or knockdown of the TFs in the cells before their differentiation into pDCs. However, it may be constrained by suboptimal HSC transduction and the efficiency of the chosen RNAi effectors.

The dynamics among pDC lineage-specific TFs control the development and function of pDCs. Although debate persists regarding their ontogeny, and support for a lymphoid origin has recently been presented (47–51), it has been proposed that pDCs may develop from both common dendritic cell progenitors (CDPs) and common lymphoid progenitors. A recent investigation into the transcriptional regulatory program of DCs in mice suggested that, while cDCs follow the default developmental program from CDPs, pDCs differentiate through a subnetwork that requires reinforcement mechanisms and several regulatory feedback loops between various TFs to stabilize the pDC program (52). BCL11A is widely expressed in HSCs and directs the commitment of CDPs to the pDC lineage by regulating the transcription levels of TCF4 and its antagonist ID2. In mice, a feedback loop between BCL11A and TCF4 has been described, which maintains homeostasis within the pDC population (53). Furthermore, the recruitment of BCL11A to various pDC related factors, including SPI1, SPIB, TCF4, and ID2, has been documented in the CAL-1 human pDC cell line (53). The balance between the TFs TCF4 and ID2 has been reported to drive the differentiation of CDPs into pDCs or cDCs subsets, respectively. TCF4, also known as E2-2, is highly expressed in pDCs, where it activates the pDC-specific gene expression program and is essential for maintaining lineage identity in mature pDCs in both mice and humans (33, 54). Genes conventionally linked to pDCs, including LILRA4 and PACSIN1, as well as the TFs BCL11A, SPIB, IRF7, and RUNX2, and components of the TLR signaling pathway (TLR9, and TLR7/TLR8), have been identified as TCF4 binding targets in human pDC cell lines (27, 54). SPIB and IRF8 are essential for the survival and functionality of pDCs. SPIB promotes pDC survival by suppressing apoptosis through the induction of the antiapoptotic gene BCL2A1, essential for human pDC development (55). IRF8 plays a crucial role in the development of conventional type 1 dendritic cells (cDC1s) and pDCs. Two trajectories dependent on IRF8 dosage have been proposed: the IRF8^Hi^ pathway, shared by cDC1s and pDCs, and the IRF8^Lo^ pathway, associated with monocytes (56). Individuals with biallelic mutations in IRF8 show a complete absence of monocytes and dendritic cells (56). Additionally, it has been observed in mice that the loss of IRF8 impairs the ability of pDCs to produce type I IFNs but enhances their antigen presentation capacity (57). The impaired HSPC-pDC differentiation and functionality we observed when knocking out BCL11A, TCF4, SPIB, and IRF8 in HSPCs aligns with these previous findings in murine and human pDCs and highlights the significance of these TFs in pDC biology. Furthermore, the opposing effect on the phenotype of HSPC-pDCs resulting from the knockout of ID2, a TF that antagonizes TCF4, supports this assertion.

In our analysis, we also found two TFs, CUX2, and ZFAT, among the top 50 most significant DEGs between CD123H and DPos subsets. Both TFs have been associated with pDCs in previous studies, but their roles in pDC function remain unknown (29, 30, 32). In mice, ZFAT has been proposed to contribute to the control of common aspects of pDC, cDC1, and cDC2 development (58). Furthermore, the MYB-ZFAT gene fusion has been identified in patients with blastic pDC neoplasm, a hematological malignancy derived from pDC precursors (59). These findings suggest potentially significant roles for CUX2 and ZFAT in pDC biology. Our data knocking these two TFs out indicated a compromised expansion during HSPC-pDC differentiation, but neither the phenotype nor the TLR9 responsiveness of the cells were affected by loss of these TFs. Therefore, their potential role in pDC biology remains elusive and warrants further investigation.

To summarize, we identified a subset of cells from pDC *in vitro* differentiation, CD123H, distinguished by its elevated expression of the pDC marker CD123. The elevated expression of genes associated with the conventional blood pDC signature coupled with the increased production of IFNα upon TLR9 stimulation, suggests that this subset resembles mature blood pDCs in terms of TLR9 responsiveness. In contrast, cells expressing low to intermediate levels of CD123 contain a small subset of TLR-responsive cells as well as a subset with an immature TLR9 pathway that may be acquired during further culture. Our study focused exclusively on the TLR9 pathway, but future studies should investigate responses to other immune agonists to probe HSPC-pDC activation through other pathways such as TLR7, cGAS-STING, and RIG-I, which may unravel further functional diversity among the subsets. We have also demonstrated the feasibility of redirecting the cell fate of CD34+ HSPCs during pDC specification through genetic manipulation of key TFs, as evidenced by enhanced differentiation upon ID2 knockout resulting in increased numbers of TLR9-responsive cells. Hence, this showcases that our technological platform can probe gene-phenotype relationships in pDC differentiation and biology and suggests that precise modulation of the expression of key TFs could direct the production of distinct cell subsets with unique features in pDC differentiation cultures *in vitro*.

## Materials and methods

### Isolation of HSPCs from CB

Deidentified umbilical cord blood samples (UCB) from scheduled caesarean deliveries of healthy infants were collected at the Department of Gynecology and Obstetrics, Aarhus University Hospital, Denmark. Informed written consent was obtained from the mothers, but studies on anonymized samples, such as those used in the present study, are exempt from ethical permissions in Denmark (Komitéloven §14, stk. 3). CD34-positive HSPCs were isolated using EasySep Human Cord Blood CD34 Positive Selection Kit II (STEMCELL Technologies, Cat. No: #17896) according to the manufacturer’s instructions. The isolated CD34-positive cells were either utilized immediately or cryopreserved until needed.

### Differentiation of human HSPCs into pDCs

HSPC‐pDCs were generated using DC medium, following the procedure described in a previous publication for generating HSPC-pDCs from HSPCs (15). Briefly, HSPCs were cultured in DC medium (GMP DC Medium, Cat. No: 20801-0500, Sartorius CellGenix GmbH) at low density (2E5 – 1E6 cells/mL). For all conditions, the medium was supplemented with 50 µg/mL of ascorbic acid (Merck, Cat. No: A4403) and the cytokines and growth factors Flt3-L (100 ng/mL), SCF (100 ng/mL), TPO (50 ng/mL), and IL-3 (20 ng/mL) (Human Hematopoietic Stem Cell Expansion Cytokine Package, PeproTech, Cat. No: HHSC3). Cells were cultured at 37°C, 95% humidity, and 5% CO2 for up to 16 days. The medium was replenished every 2–4 days depending on the growth of the HSPC-pDCs.

### Priming of HSPC-pDCs

HSPC-pDC priming was carried out as previously described (14). HSPC-pDCs were cultured in DC medium supplemented with P/S, 50 µg/mL ascorbic acid (Merck, Cat. No: A4403), and 20 ng/mL IL-3 (20 ng/mL) (Human Hematopoietic Stem Cell Expansion Cytokine Package, PeproTech, Cat. No: HHSC3). For priming, pDCs were primed with 250 U/mL IFN-β (PBL Assay Science, Cat. No: 11410) and 250 U/mL IFN-γ (PeproTech, Cat. No: 300-02) for 24 hours.

### TLR9 stimulation and cytokine quantification in pDC supernatants

4×10^4 primed HSPC-pDCs were plated in 96-well flat-bottom plates in a final volume of 200 μl per well of DC medium only supplemented with P/S and IL-3 (20 ng/mL), and then stimulated with 2.5 μg/mL of agonists directed against TLR9 (CpG-A 2216, tlrl-2216-1, InvivoGen). After 20h stimulation, pDC culture supernatants were harvested and cryopreserved at –20°C until analysis. Supernatants were later measured in duplicate for cytokines IFN-L1/IL-29, IFN-α2a, IFN-β, and TNF-α, using Meso Scale Discovery (MSD, Rockville, MA, USA) multiplex assay, following manufacturer’s instructions.

### Cell surface phenotype by flow cytometry

The following fluorochrome-conjugated antibodies were used for staining cell surface markers: anti-human Lineage Cocktail (APC, BioLegend, Cat. No:348803), anti-human CD11c (APC, BioLegend, Cat. No:301614), anti-human CD123 (PE, eBioscience, clone 6H6, Cat. No:12-1239-41), anti-human CD303 (PE-Cy7, eBioscience, clone 201A, Cat. No: 25-9818-41; BV421, BioLegend, Cat. No: 354212; PE-Vio770, Miltenyi Biotec, Cat. No: 130-113-655). Cell surface pDC staining was performed in FACS buffer (PBS, 2% FBS, 1 mM EDTA) for 20 minutes at 4°C. Dead cells were excluded using the Ghost Dye Red 780 Viability Dye (Tonbo Biosciences, Cat. No: 13-0865) or the Zombie Violet Fixable Viability Kit (BioLegend, Cat. No: 423113) at a 1:100 dilution. For experiments involving blocking reagents, 2.5 µL of either True-Stain Monocyte Blocker (BioLegend, Cat. No: 426103), Human IgG (Merck, Cat. No: I4506), and/or TruStain (BioLegend, Cat. No: 422302) was added to the cells in a total volume of 50 µL of FACS buffer. The cell suspensions were incubated at room temperature for 15 minutes before proceeding with extracellular staining. No washing steps were performed between blocking and immunostaining.

### Intracellular staining for IFNα

Following TLR9 stimulation with CpG-A, brefeldin A (BioLegend, Catalog No. 420601) was added to the cell culture medium at a final concentration of 1 µl per 1 ml of medium, and the cells were incubated for an additional 2 hours before antibody staining. After staining cells for surface markers, cells were fixed in 100 µL of Fixation Buffer (Intracellular Fixation & Permeabilization Buffer Set eBioscience, Cat. No: 88-8824-00) for 20 min at room temperature, and then additionally permeabilized in 100 µL of 1X Permeabilization Buffer (Intracellular Fixation & Permeabilization Buffer Set eBioscience, Cat. No: 88-8824-00) +2 µL of FcR Blocking solution (Human TruStain FcX, BioLegend Cat. No: 422302) for 15 min at room temperature. Finally, cells were incubated for 30 minutes at 4°C with the IFN-α Antibody primary antibody (FITC, Miltenyi Biotec, Clone LT27:295, Cat. No: 130-128-082) diluted in 1X Permeabilization Buffer at the recommended dilution (1:50). Cell debris and dead cells were excluded from the analysis.

### Cell sorting and RNA-seq

HSPC-pDCs were stained for pDC surface markers as detailed in the previous section, and subsequently sorted using a FACSAria III flow cytometer equipped with four lasers (405, 488, 561, and 633 nm) and twelve fluorescence detectors (BD Biosciences, Franklin Lakes, NJ, USA). The acquisition was performed using BD FACSDiva software v.8.0.2. (BD Biosciences). Total RNA from sorted HSPC-pDCs was extracted using the ReliaPrep RNA Cell Miniprep System (Promega, Cat. No: Z6012), following the manufacturer’s instructions. Total RNA was sent to BGI Europe for RNA-seq. Here, a non-stranded and poly(A)-enriched mRNA library was constructed from total RNA, which was subsequently subjected to PE100 sequencing on the BGISEQ platform.

### RT-qPCR

Total RNA (100 ng) was reverse-transcribed using iScript Reverse Transcription Supermix for RT-qPCR (Bio-Rad Laboratories, Cat. No: 1708840) and stored at −20°C. RT-qPCR amplification was performed using Takyon™ No ROX SYBR 2X MasterMix blue dTTP (Eurogentec, Cat. No: UF-NSMT-B0701) on a LightCycler^®^ 480 Instrument II (Roche Diagnostics). The thermal cycling profile consisted of initial denaturation at 95°C for 3 min, followed by 40 cycles of denaturation at 95°C for 10 s, annealing at 60°C for 20 s, and extension at 72°C for 20 s. Relative expression levels were determined using the 2-ΔΔCt method and normalized to β-ACT as the reference gene. The primers used for RT-qPCR were as follows: β-ACT Fw, 5´-CCTTCCTGGGCATGGAGT-3´; β-ACT Rv, 5´-GGAGCAATGATCTTGATCTTC-3´; IFNA-1/13 Fw, 5´-CCAGTTCCAGAAGGCTCCAG-3´; IFNA-1/13 Rv, 5´-TGCATCACACAGGCTTCCAA-3´.

### Assembly of RNP complexes and nucleofection

The formation of RNP was done by mixing chemically synthesized sgRNAs (**Supplementary Table 3**) obtained from Synthego (Silicon Valley, CA, USA) with the Alt-R S.p. Cas9 Nuclease V3 (IDT, Coralville, IA, USA) at a molar ratio of 1:2.5, and incubated at 25°C for 15 minutes. Then, RNP complexes were delivered to cells by nucleofection. For the nucleofection, 10^5 HSPCs were electroporated using the Lonza 4D-Nucleofector device (Lonza Bioscience) and 20 μL Nucleocuvette Strips, using 1M solution and applying the DZ100 program. As controls, HSPCs either electroporated without RNPs (Mock) or RNPs targeting the AAVS1 locus were included in all experiments. Editing efficiency for each target was quantified using the Synthego ICE analysis tool. PCR was performed using primers flanking the edited region, and Sanger sequencing of the PCR products was subsequently conducted.

### Statistical analysis

All data were plotted using GraphPad Prism 10 (GraphPad Software, San Diego, CA). The data are presented as means of biological replicates ± standard error of the mean (± SEM), or as means ± standard deviation (SD) in samples consisting of only two replicates. Statistically significant differences between groups were determined using one-way ANOVA, followed by Bonferroni’s *post-hoc* test, or the Kruskal-Wallis test when the assumptions for one-way ANOVA were not met. *p < 0.05, **p < 0.01, ***p < 0.0001.

## Data Availability

The raw RNA-seq files from this study are available in the BioProject database (BioProject ID: PRJNA1226126). For additional information or requests for reagents, please contact the corresponding author.
