# Corrigendum: Characterization of TLR9 responsiveness in cell subsets derived from *in vitro* pDC differentiation of hematopoietic stem and progenitor cells

**DOI:** 10.3389/fimmu.2025.1625107

**Published:** 2025-05-29

**Authors:** Sabina Sánchez Hernández, Tobias Wang Bjerg, Ian Helstrup Nielsen, Anders Laustsen, Hai Q Tang, Lars Henning Pedersen, Eynav Klechevsky, Martin R. Jakobsen, Rasmus O. Bak

**Affiliations:** ^1^ Department of Biomedicine, Aarhus University, Aarhus, Denmark; ^2^ Department of Clinical Medicine, Aarhus University, Aarhus, Denmark; ^3^ Department of Obstetrics and Gynaecology, Aarhus University Hospital, Aarhus, Denmark; ^4^ Department of Pathology and Immunology, Division of Immunobiology, Washington University School of Medicine, St. Louis, MO, United States

**Keywords:** plasmacytoid dendritic cells, CD34 hematopoietic stem cells, *in vitro* differentiation, subsets, type I IFN

In the published article, there was an error in **Figure 8B** as published. **Figure 8** has been updated. The revised file restores axis label in **Figure 8B**, which was lost during the article production process. The scientific content remains intact, and the revision ensures the figure is presented as originally intended.

**Figure 8:**
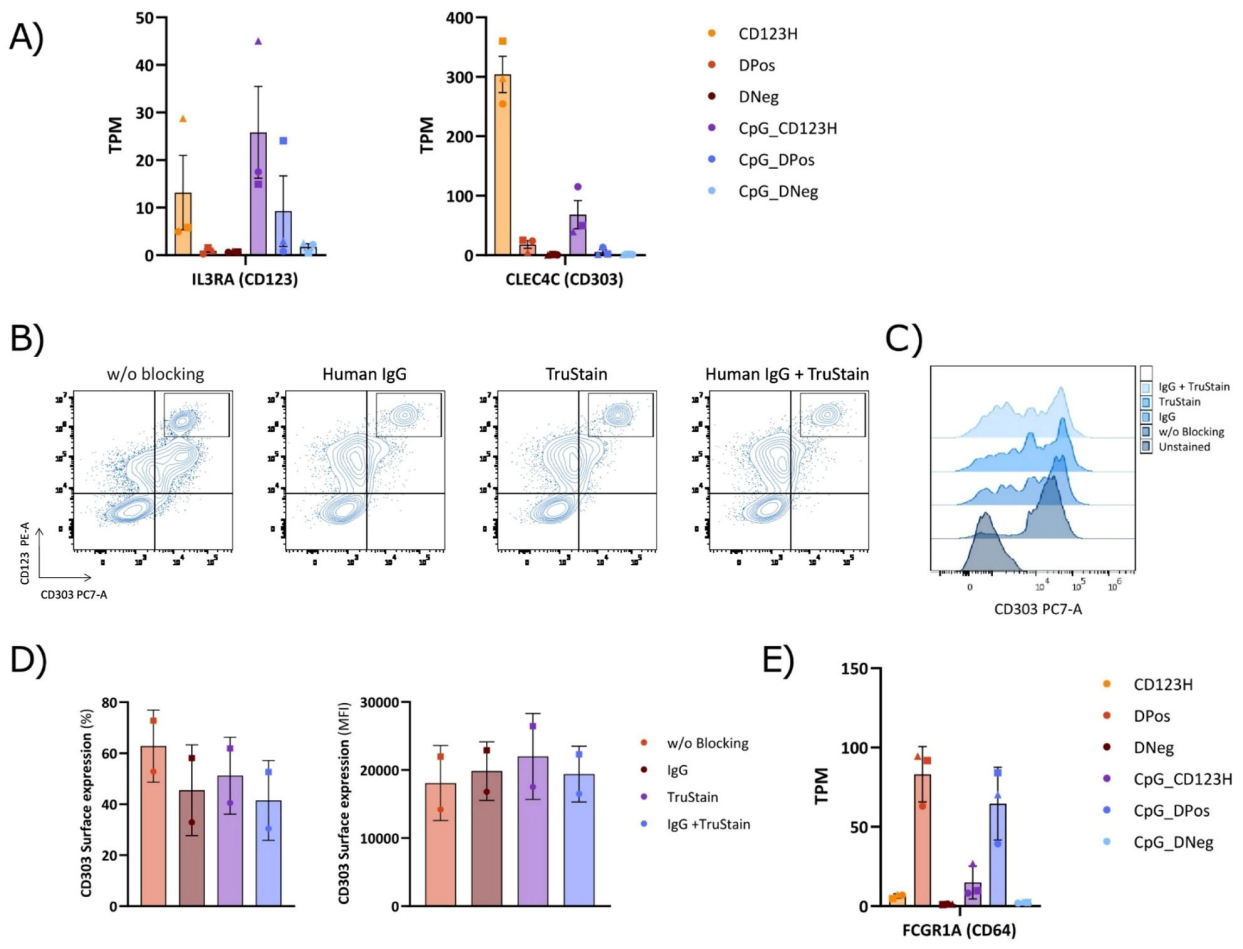
Comparison of Fc receptor blocking methods in the cell surface staining of subsets derived from HSPC-to-pDC differentiation. **(A)** Bar graph displaying Transcripts Per Million (TPM) counts of the human pDC markers CD123 (IL3RA) and CD303 (CLEC4C) in each subset. **(B)** Representative flow cytometry plots showing the cell surface expression of CD123 and CD303 in cells on day 16 of differentiation, employing human IgG alone, TruStain alone, or a combined blocking approach. **(C)** Flow cytometry histograms illustrating the CD303 expression profile obtained following the indicated blocking method. **(D)** Bar graphs showing the proportion of CD303 positive cells within hLin and CD11c negative cells (left) and the CD303 surface expression levels (MFI) (right) using various blocking methods during surface staining. Data are presented as mean ± SD from two donors. **(E)** Bar graph showing Transcripts Per Million (TPM) counts of the Fc gamma receptor I (Fc γRI; FCGR1A).

In the published article, some graphical elements, such as text boxes and axes, were inadvertently omitted of **Supplementary Figures S1A**, **S5**, and **S6** during the production process. The missing material has been updated in the original article.

The authors apologize for these errors and state that they do not change the scientific conclusions of the article in any way. The original article has been updated.

