# Supplementary material for: Characterization of TLR9 responsiveness in cell subsets derived from in vitro pDC differentiation of hematopoietic stem and progenitor cells
Source: Front Immunol. 2025 Mar 27;16:1550397. doi: 10.3389/fimmu.2025.1550397 (PMC11983628; doi:10.3389/fimmu.2025.1550397)
Supplement: Supplementary file 1 [file DataSheet1.pdf]

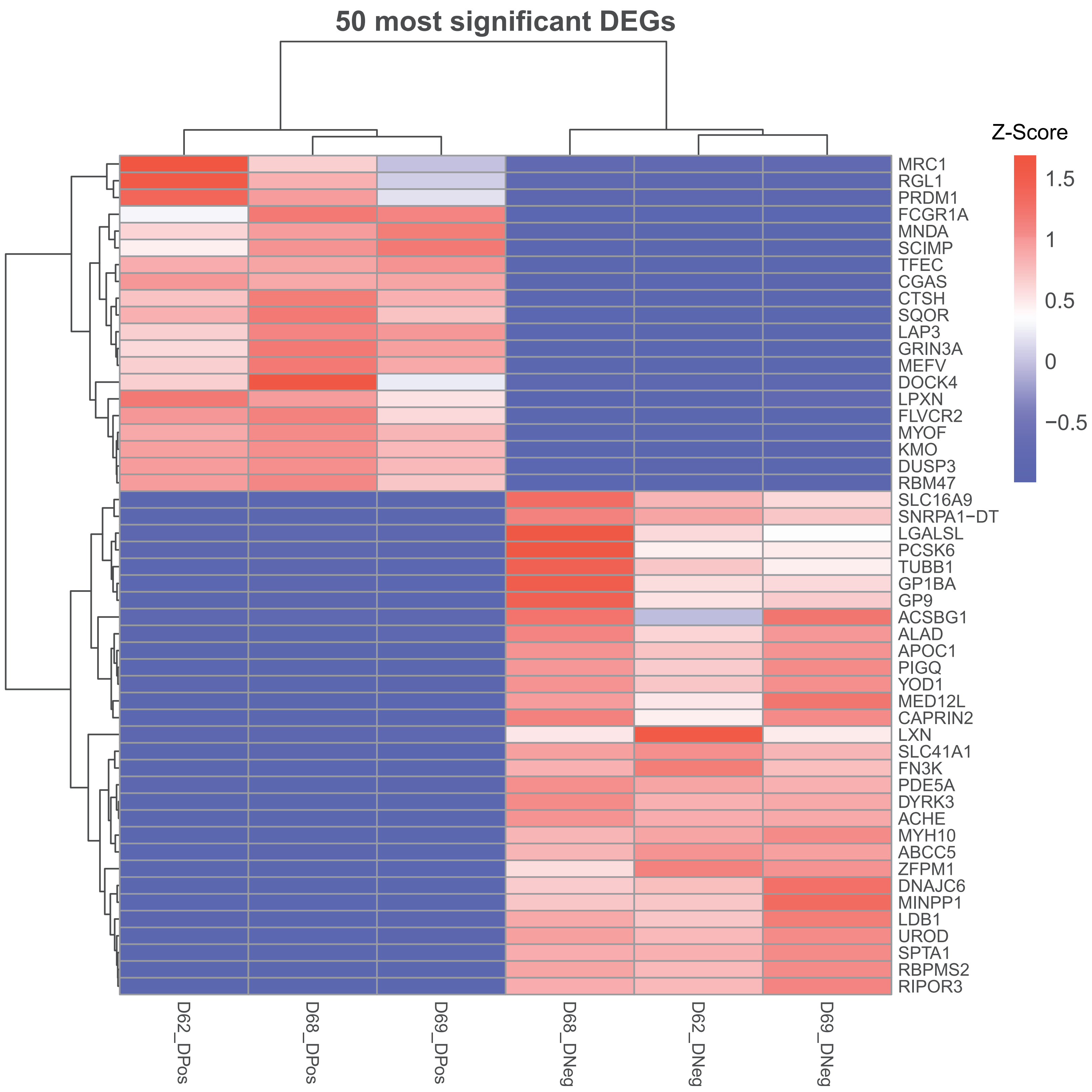
50 most significant DEGs
Z-Score
1.5
1
0.5
0
−0.5
MRC1
RGL1
PRDM1
FCGR1A
MNDA
SCIMP
TFEC
CGAS
CTSH
SQOR
LAP3
GRIN3A
MEFV
DOCK4
LPXN
FLVCR2
MYOF
KMO
DUSP3
RBM47
SLC16A9
SNRPA1−DT
LGALSL
PCSK6
TUBB1
GP1BA
GP9
ACSBG1
ALAD
APOC1
PIGQ
YOD1
MED12L
CAPRIN2
LXN
SLC41A1
FN3K
PDE5A
DYRK3
ACHE
MYH10
ABCC5
ZFPM1
DNAJC6
MINPP1
LDB1
UROD
SPTA1
RBPMS2
RIPOR3
D62_DPos
D68_DPos
D69_DPos
D68_DNeg
D62_DNeg
D69_DNeg

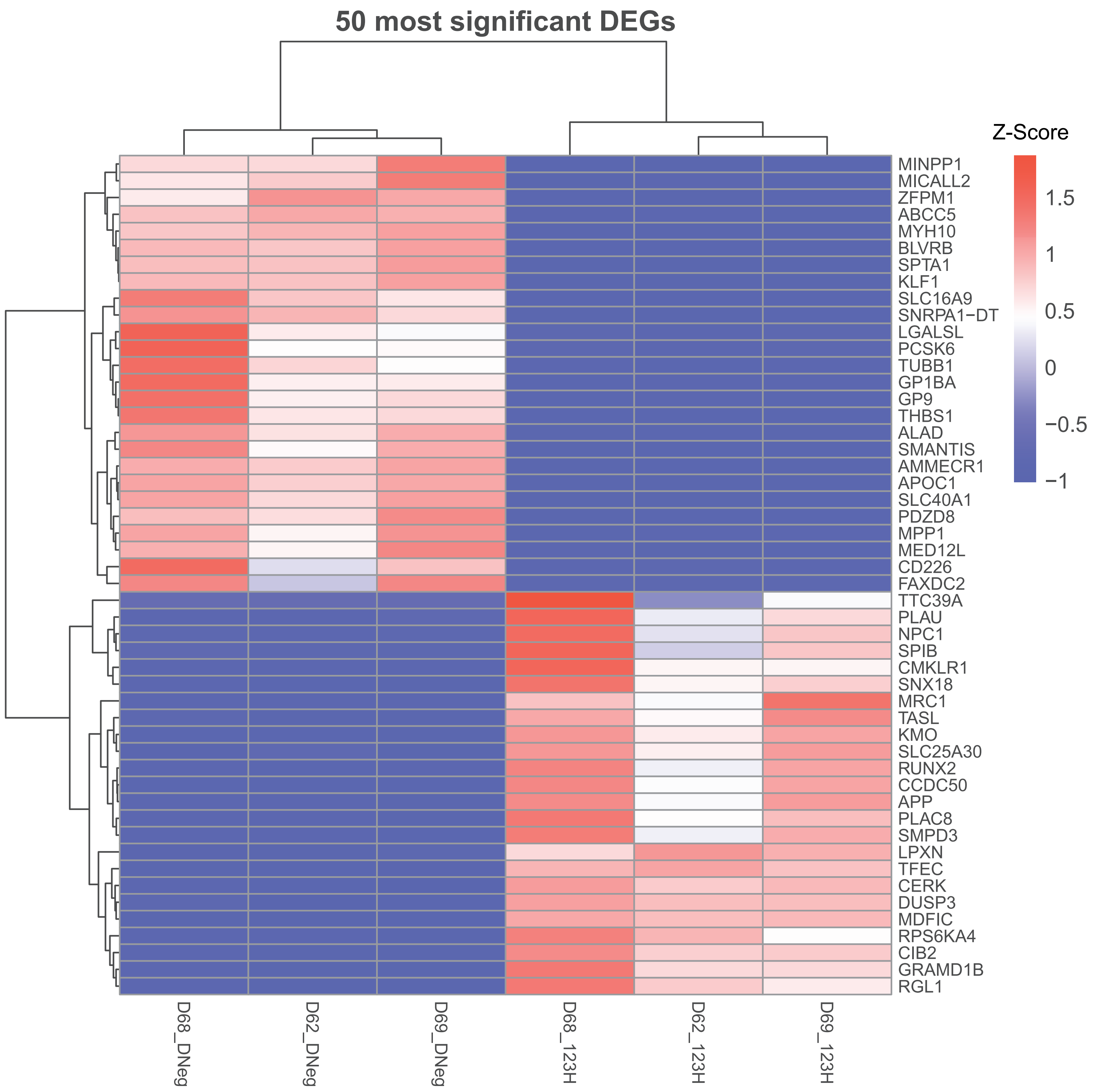

50 most significant DEGs
Z-Score
1.5
1
0.5
0
−0.5
−1
MINPP1
MICALL2
ZFPM1
ABCC5
MYH10
BLVRB
SPTA1
KLF1
SLC16A9
SNRPA1−DT
LGALSL
PCSK6
TUBB1
GP1BA
GP9
THBS1
ALAD
SMANTIS
AMMECR1
APOC1
SLC40A1
PDZD8
MPP1
MED12L
CD226
FAXDC2
TTC39A
PLAU
NPC1
SPIB
CMKLR1
SNX18
MRC1
TASL
KMO
SLC25A30
RUNX2
CCDC50
APP
PLAC8
SMPD3
LPXN
TFEC
CERK
DUSP3
MDFIC
RPS6KA4
CIB2
GRAMD1B
RGL1
D68_DNeg
D62_DNeg
D69_DNeg
D68_123H
D62_123H
D69_123H

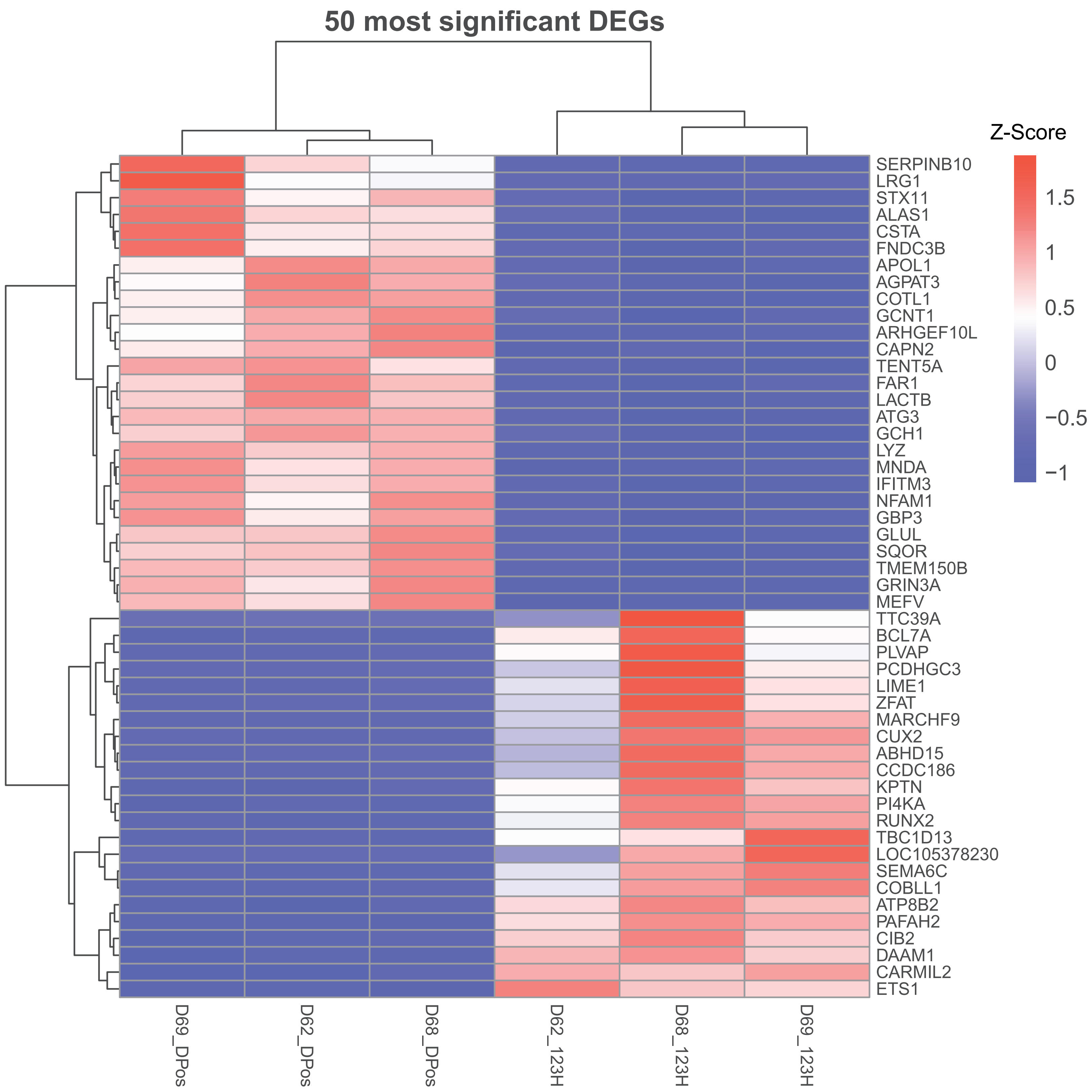

50 most significant DEGs
Z-Score
1.5
1
0.5
0
−0.5
−1
SERPINB10
LRG1
STX11
ALAS1
CSTA
FNDC3B
APOL1
AGPAT3
COTL1
GCNT1
ARHGEF10L
CAPN2
TENT5A
FAR1
LACTB
ATG3
GCH1
LYZ
MNDA
IFITM3
NFAM1
GBP3
GLUL
SQOR
TMEM150B
GRIN3A
MEFV
TTC39A
BCL7A
PLVAP
PCDHGC3
LIME1
ZFAT
MARCHF9
CUX2
ABHD15
CCDC186
KPTN
PI4KA
RUNX2
TBC1D13
LOC105378230
SEMA6C
COBLL1
ATP8B2
PAFAH2
CIB2
DAAM1
CARMIL2
ETS1
D69_DPos
D62_DPos
D68_DPos
D62_123H
D68_123H
D69_123H

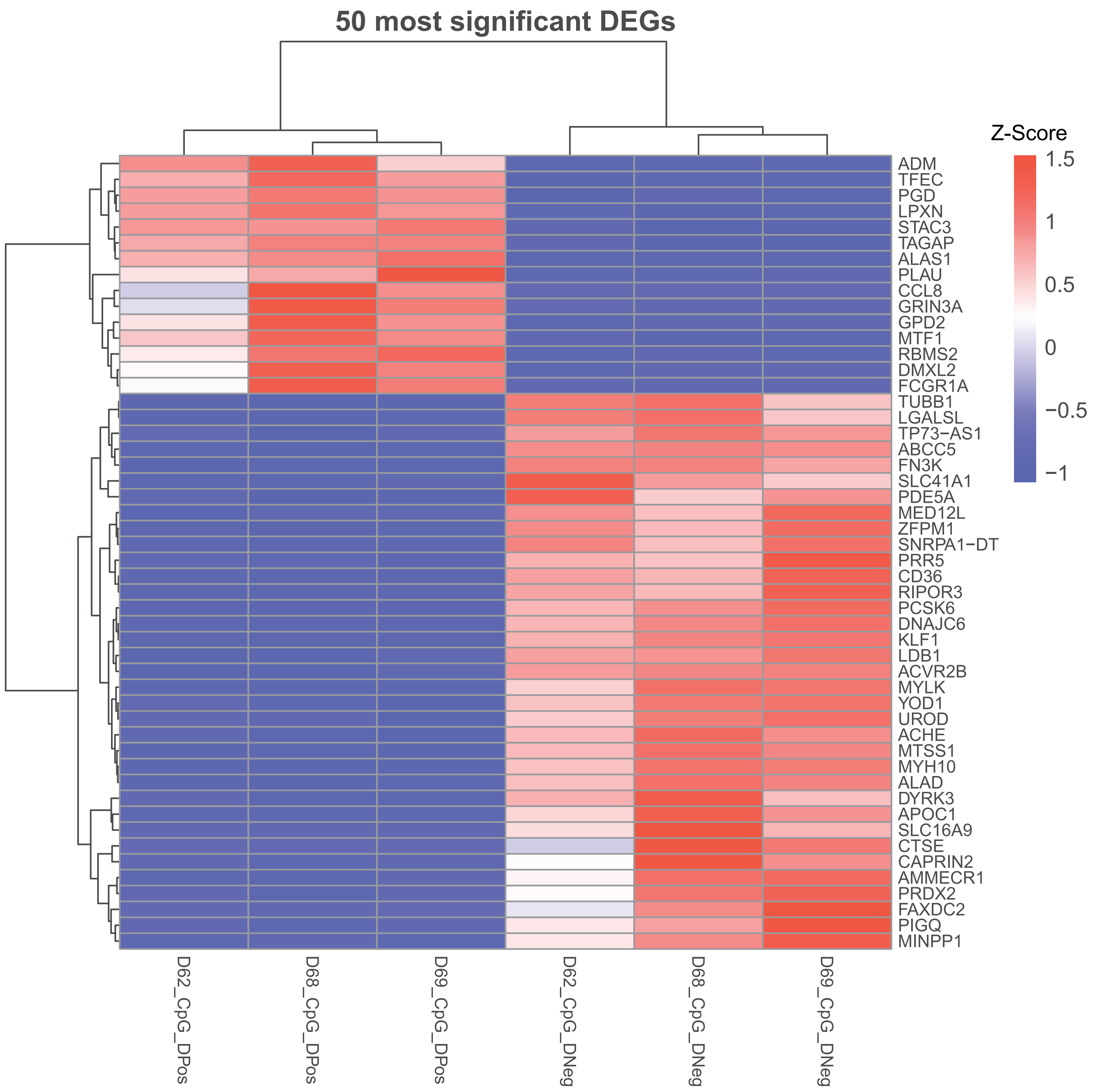
50 most significant DEGs
Z-Score
1.5
1
0.5
0
−0.5
−1
ADM
TFEC
PGD
LPXN
STAC3
TAGAP
ALAS1
PLAU
CCL8
GRIN3A
GPD2
MTF1
RBMS2
DMXL2
FCGR1A
TUBB1
LGALSL
TP73−AS1
ABCC5
FN3K
SLC41A1
PDE5A
MED12L
ZFPM1
SNRPA1−DT
PRR5
CD36
RIPOR3
PCSK6
DNAJC6
KLF1
LDB1
ACVR2B
MYLK
YOD1
UROD
ACHE
MTSS1
MYH10
ALAD
DYRK3
APOC1
SLC16A9
CTSE
CAPRIN2
AMMECR1
PRDX2
FAXDC2
PIGQ
MINPP1
D62_CpG_DPos
D68_CpG_DPos
D69_CpG_DPos
D62_CpG_DNeg
D68_CpG_DNeg
D69_CpG_DNeg

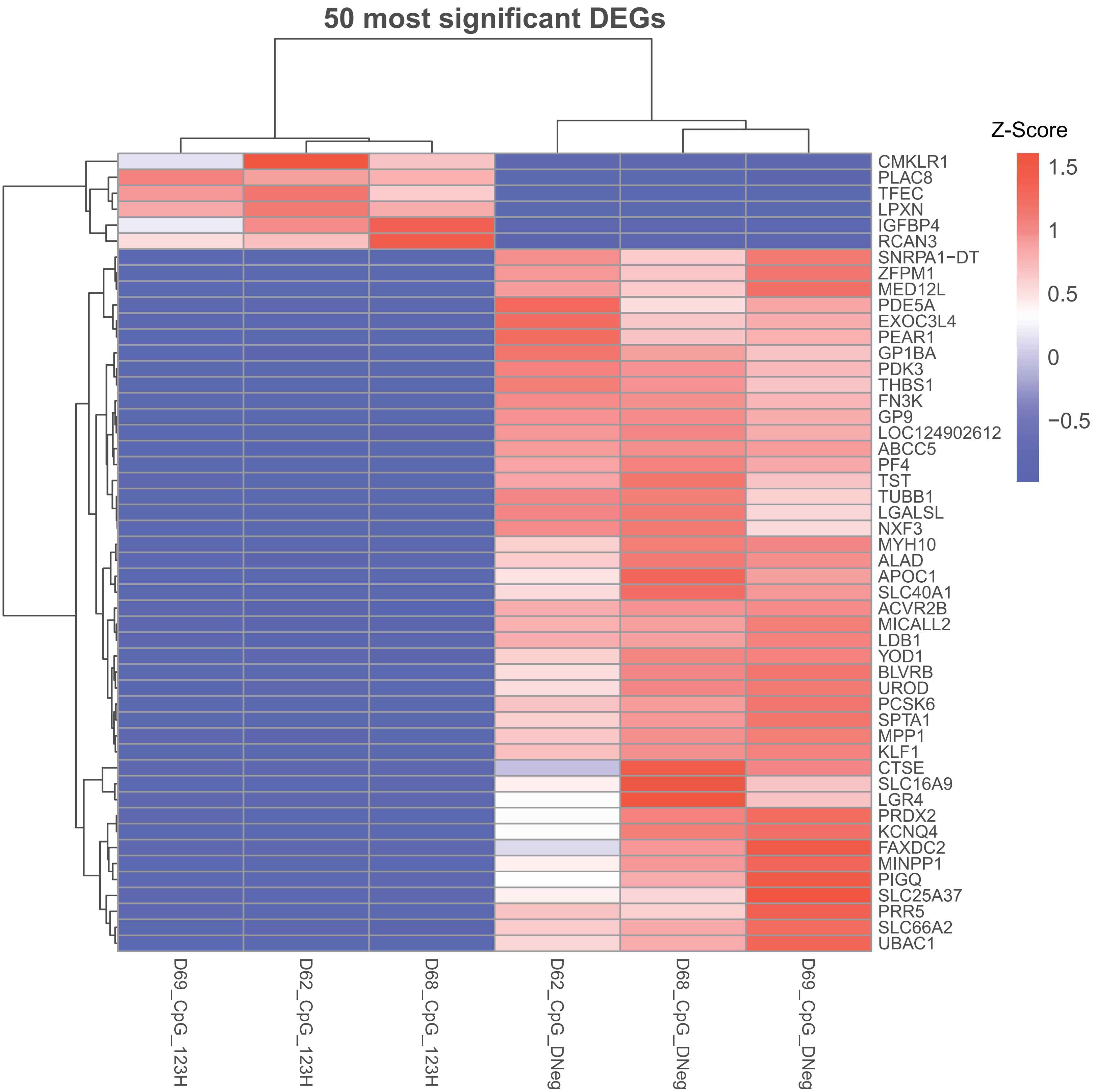
50 most significant DEGs
Z-Score
1.5
1
0.5
0
−0.5
CMKLR1
PLAC8
TFEC
LPXN
IGFBP4
RCAN3
SNRPA1−DT
ZFPM1
MED12L
PDE5A
EXOC3L4
PEAR1
GP1BA
PDK3
THBS1
FN3K
GP9
LOC124902612
ABCC5
PF4
TST
TUBB1
LGALSL
NXF3
MYH10
ALAD
APOC1
SLC40A1
ACVR2B
MICALL2
LDB1
YOD1
BLVRB
UROD
PCSK6
SPTA1
MPP1
KLF1
CTSE
SLC16A9
LGR4
PRDX2
KCNQ4
FAXDC2
MINPP1
PIGQ
SLC25A37
PRR5
SLC66A2
UBAC1
D69_CpG_123H
D62_CpG_123H
D68_CpG_123H
D62_CpG_DNeg
D68_CpG_DNeg
D69_CpG_DNeg

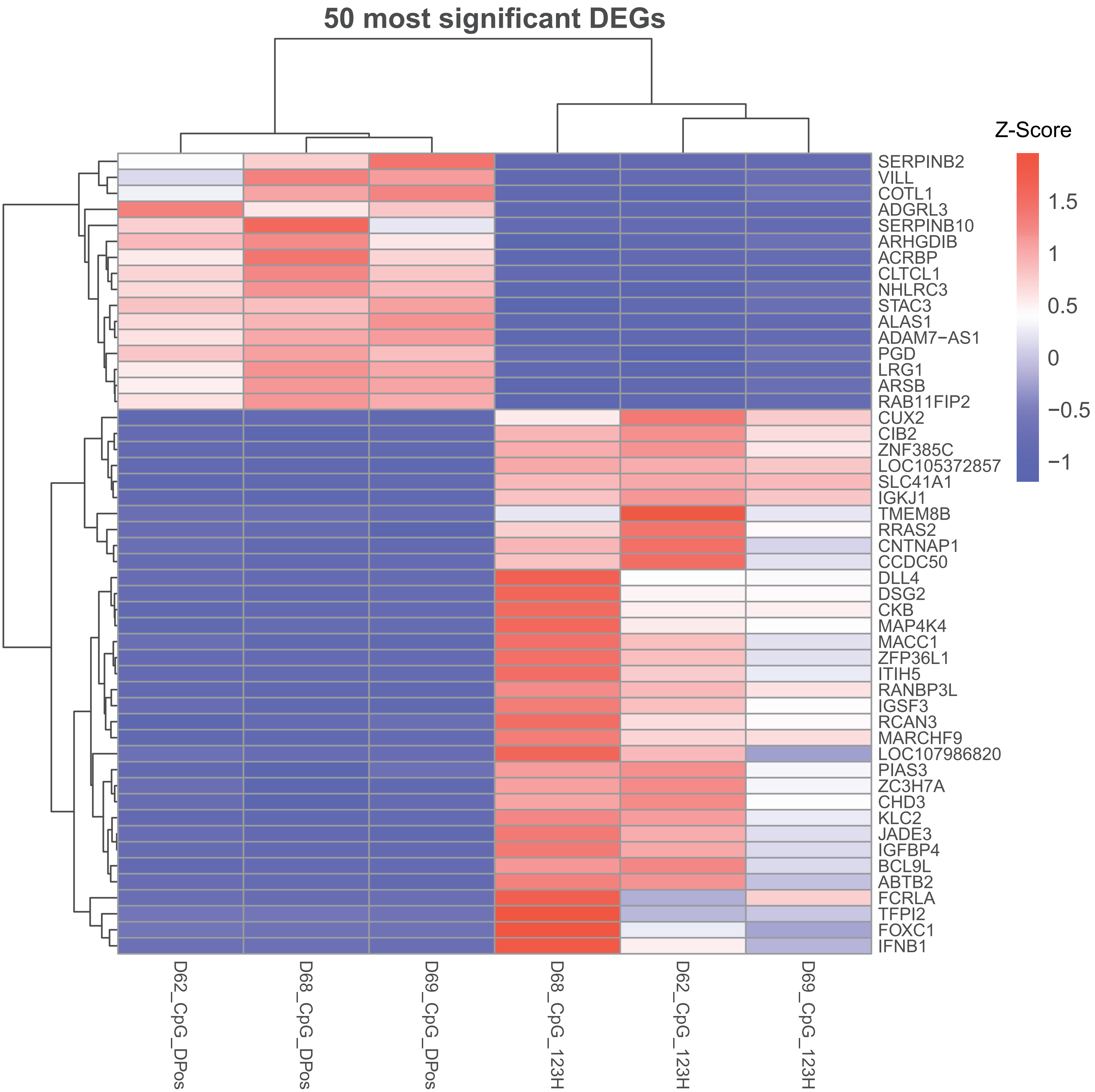

50 most significant DEGs
Z-Score
1.5
1
0.5
0
−0.5
−1
SERPINB2
VILL
COTL1
ADGRL3
SERPINB10
ARHGDIB
ACRBP
CLTCL1
NHLRC3
STAC3
ALAS1
ADAM7−AS1
PGD
LRG1
ARSB
RAB11FIP2
CUX2
CIB2
ZNF385C
LOC105372857
SLC41A1
IGKJ1
TMEM8B
RRAS2
CNTNAP1
CCDC50
DLL4
DSG2
CKB
MAP4K4
MACC1
ZFP36L1
ITIH5
RANBP3L
IGSF3
RCAN3
MARCHF9
LOC107986820
PIAS3
ZC3H7A
CHD3
KLC2
JADE3
IGFBP4
BCL9L
ABTB2
FCRLA
TFPI2
FOXC1
IFNB1
D62_CpG_DPos
D68_CpG_DPos
D69_CpG_DPos
D68_CpG_123H
D62_CpG_123H
D69_CpG_123H

**Document S1.** Heatmaps showing the 50 most differentially expressed genes among the subsets of HSPC-pDCs. Red represents a high expression level, and blue represents a low expression level. Scale is a function that normalizes gene expression data by converting it into Z-scores. Z-scores are computed as: $Z=(X-\mu)/\sigma$ where $X$ represents the counts, $\mu$ is the mean, and $\sigma$ is the standard deviation of the counts for each gene.
