# Supplementary material for: Characterization of TLR9 responsiveness in cell subsets derived from in vitro pDC differentiation of hematopoietic stem and progenitor cells
Source: Front Immunol. 2025 Mar 27;16:1550397. doi: 10.3389/fimmu.2025.1550397 (PMC11983628; doi:10.3389/fimmu.2025.1550397)
Supplement: Supplementary file 2 [file DataSheet2.pdf]

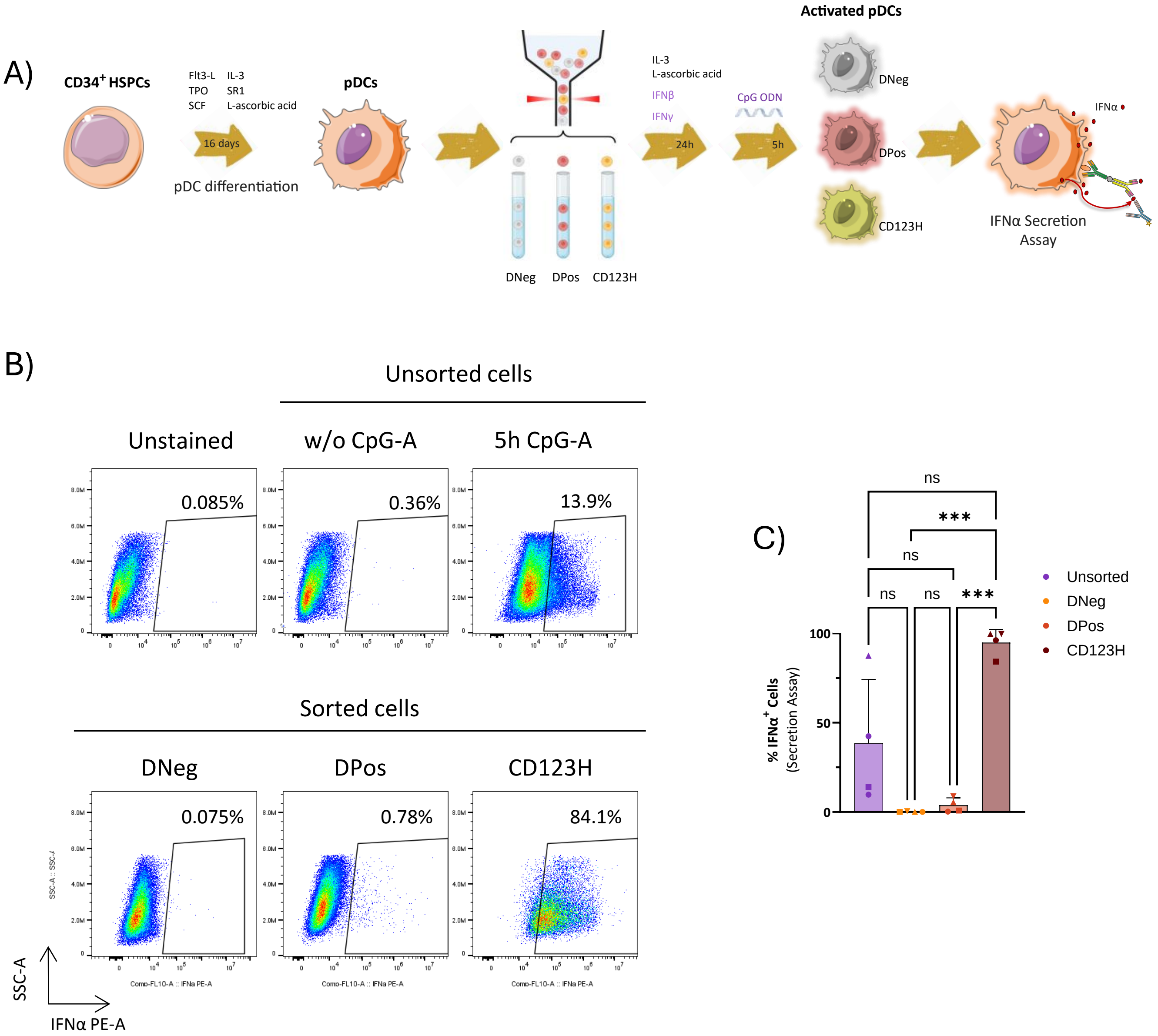

Figure S1. IFNα secretion assay performed on sorted HSPC-pDC subsets. **(a)** Schematic diagram illustrating the study design. After 16 days of HSPC-to-pDC differentiation, DNeg, DPos, and CD123H HSPC-pDC subsets were sorted. The cells were then primed with IFN-β and IFN-γ for 24 hours, followed by activation with the TLR9 agonist CpG-A for 5 hours. Subsequently, an IFNα secretion capture assay was performed in which an IFNα-specific Catch reagent is attached to the cell surface. The cells are then incubated at 37°C for 20 min to facilitate IFNα secretion. To minimize background signal where bystander cells capture IFNα from nearby IFNα-producing cells, incubation is performed at a low cell density (10^5 cells/ml). The secreted IFNα subsequently bind to the IFNα Catch Reagent on the secreting cells. These cells are then labeled with a PE-conjugated secondary IFNα-specific antibody for detection via flow cytometry. **(b)** Representative flow plots of stained HSPC-pDCs. **(c)** Graph of the sorted HSPC-pDCs and the percentage of cells that have captured IFNα on the surface. Unsorted cells were included in the analysis as a control. The data shown represent the mean ± SEM of HSPC-pDCs from four donors. One-way ANOVA was used to analyze differences between groups.

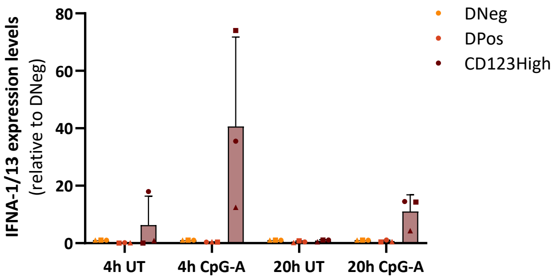

Figure S2. Relative gene expression levels of IFNA-1/13 determined by RT-qPCR in HSPC-pDCs sorted at 4 and 20 hours after TLR9 stimulation with CpG-A. Expression levels were normalized to β-actin and are shown relative to the DNeg subset. The data shown represent the mean ± SEM of sorted HSPC-pDCs from three donors

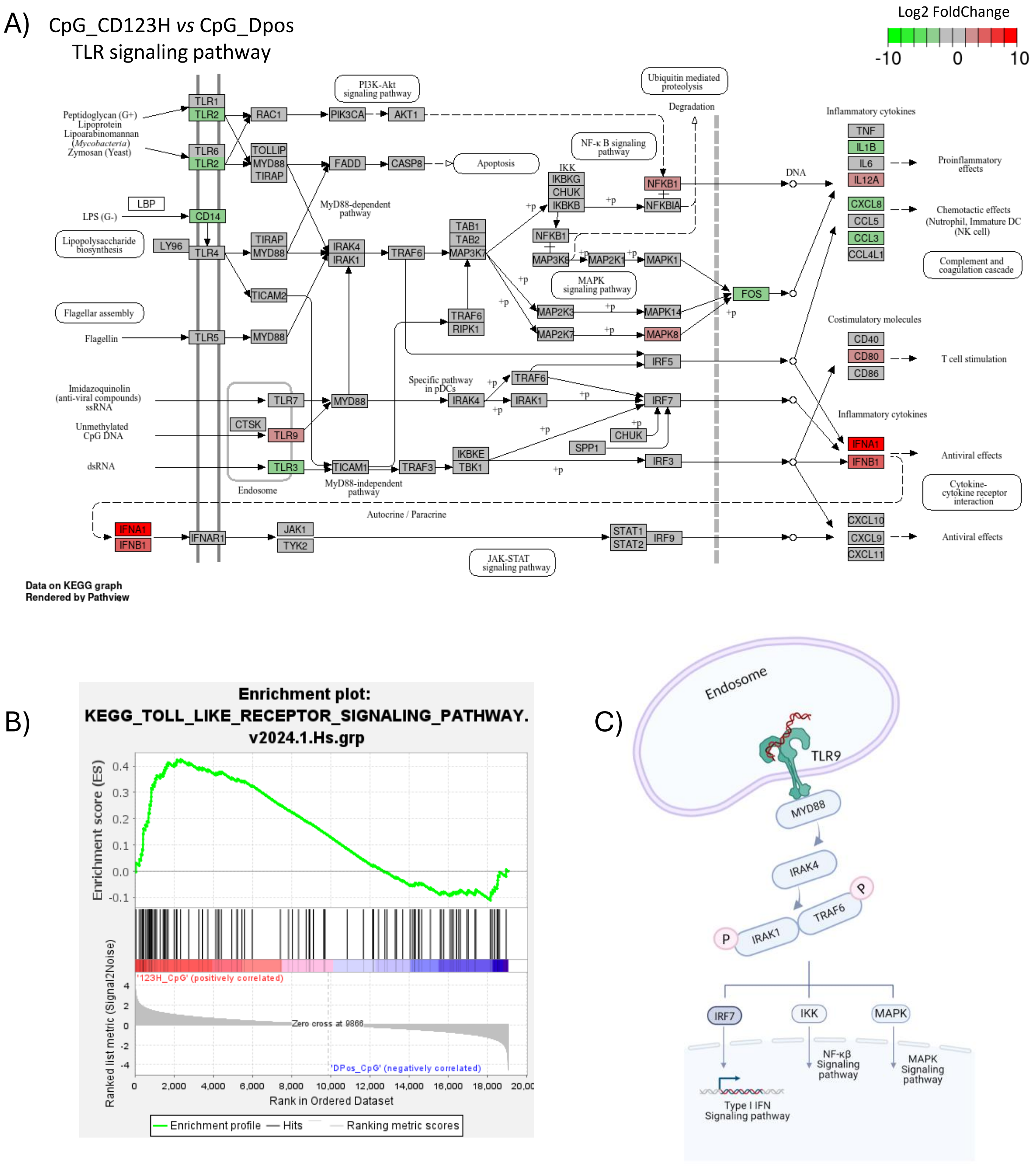

Figure S3. (a) KEGG Pathview graph of the Toll-like receptor signaling pathway (hsa04620) with differential gene expression levels shown for the CD123H subset relative to the DPos subset after 12 hours of TLR9 stimulation with CpG. **(b)** Gene Set Enrichment Analysis (GSEA) comparing the TLR signaling pathway (hsa04620) in CD123H and DPos subsets after CpG-A stimulation. Enrichment scores are shown, reflecting the degree to which this pathway is enriched in both subsets. **(c)** Simplified diagram of the signaling cascades initiated by TLR9. Binding of bacterial or viral DNA with unmethylated CpG motifs to TLR9 induces a conformational change in the receptor, facilitating the recruitment of the adaptor molecule MyD88. MyD88 then interacts with IRAK4 via its N-terminal domain. IRAK4 phosphorylates and activates IRAK1, which subsequently activate the E3 ubiquitin ligase TRAF6. This activation triggers the Type I IFN, NF-kB, and MAPK signaling pathways.

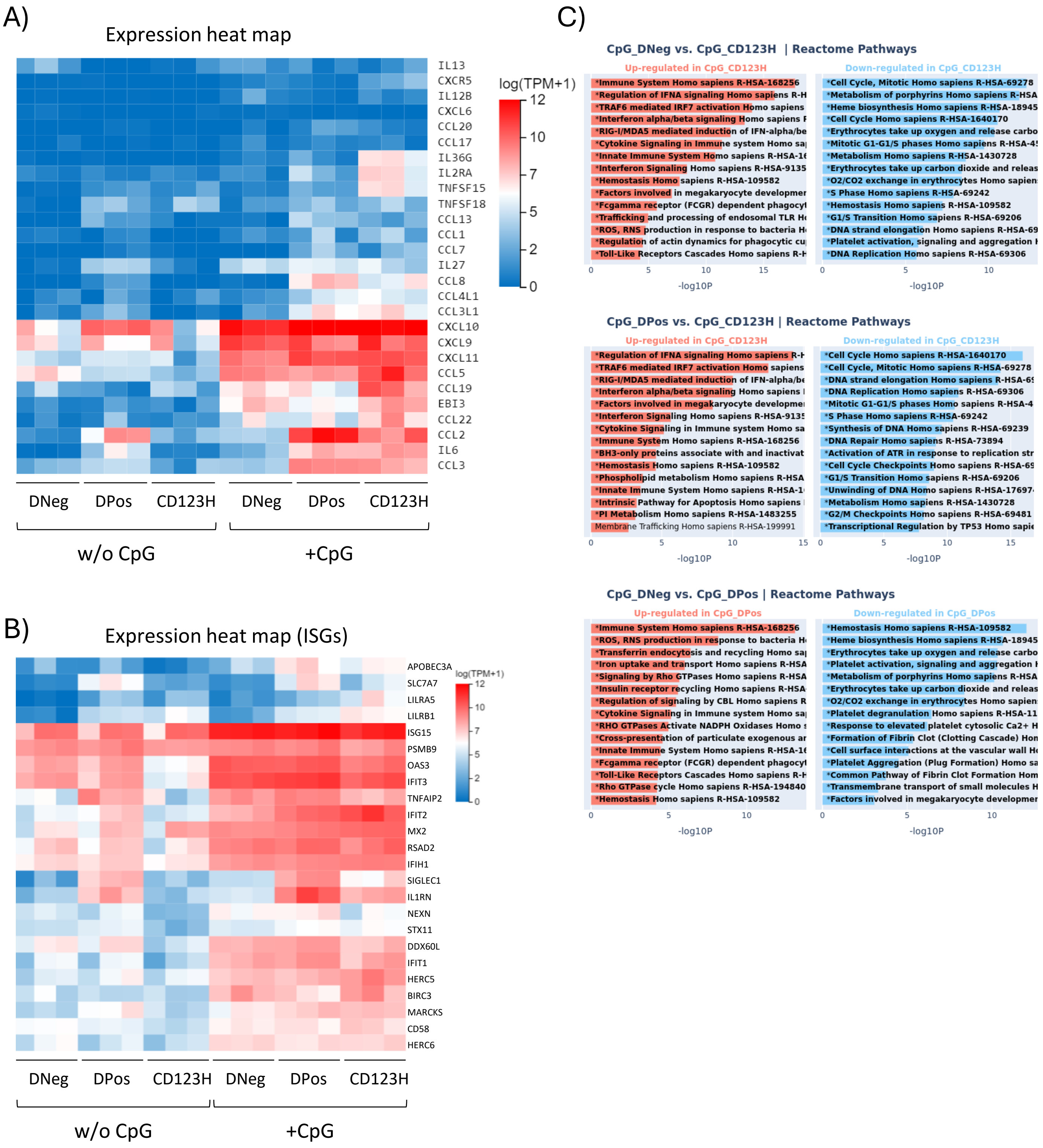

Figure S4. **(a)** Heat map displaying RNA-Seq expression data for cytokines across the three subsets of HSPC-pDCs. The panel includes the 10 cytokines most significantly upregulated in all three subsets following CpG-A stimulation. **(b)** Heat map illustrating RNA-Seq expression data for interferon-stimulated genes (ISGs) within the three sorted subsets of HSPC-pDCs. **(c)** Enrichment analysis results for HSPC-pDC subsets in Reactome Pathways. The figure contains bar charts displaying the results of the pathway enrichment analysis generated using Enrichr. The x axis indicates the -log10(P-value) for each term. Significant terms are highlighted in bold.

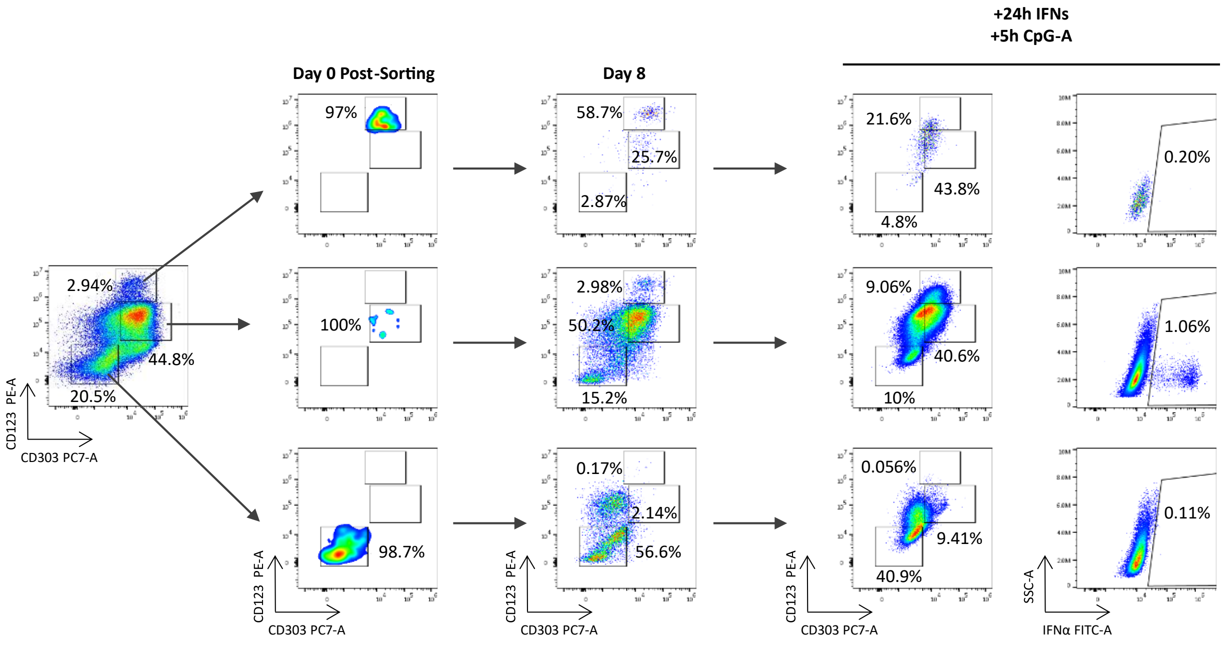

Figure S5. Differentiation Potential of HSPC-pDC Subsets from Donor 67. Flow cytometry plots showing CD123 and CD303 expression in bulk HSPC-pDCs before sorting (left), and in sorted HSPC-pDCs immediately after sorting, after 8 additional days of differentiation, and following priming + TLR9 stimulation (middle panels). The right panels show IFN-α expression in sorted HSPC-pDCs derived from donor 67 after 8 additional days of culture and subsequent TLR9 stimulation.

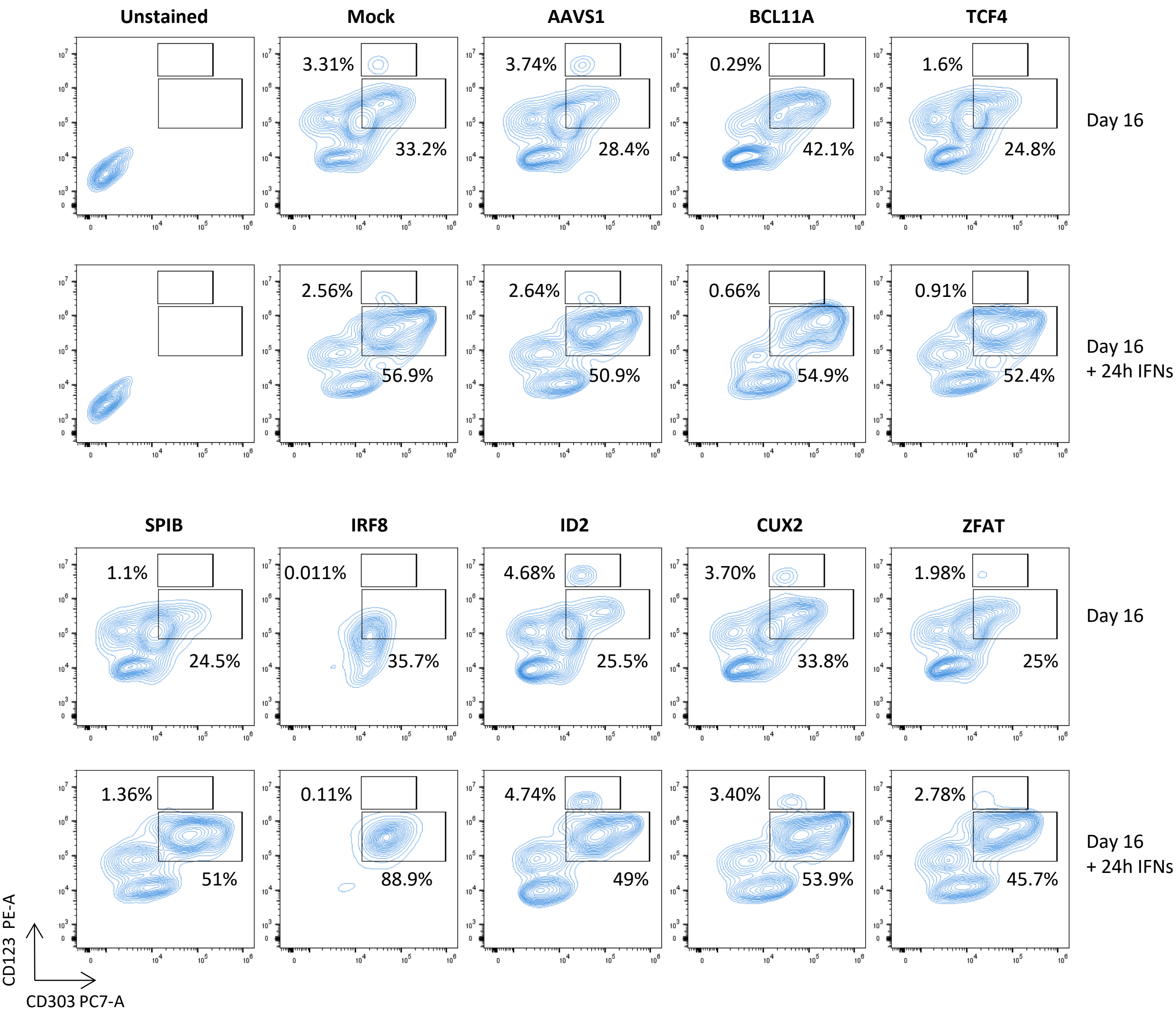

Figure S6. Representative flow cytometry plots showing cell surface expression of CD123 and CD303 in gene-edited HSPC-pDCs after 16 days of differentiation or following an additional 24-hour priming with IFN-β and IFN-γ.

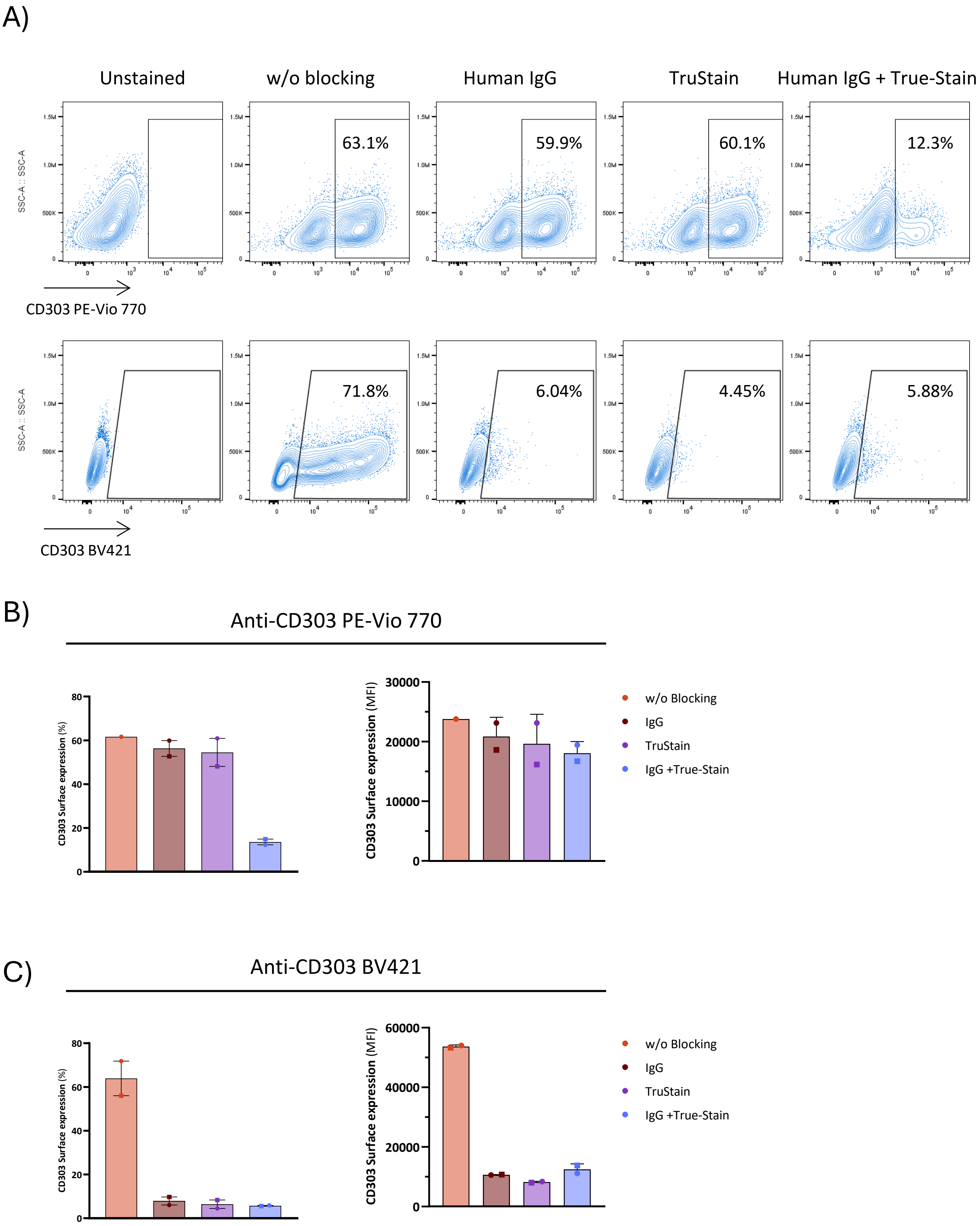

Figure S7. Comparison of CD303 expression in HSPC-pDCs using PE-Vio770- and BV421-conjugated anti-CD303 antibodies and different blocking methods. (a) Representative flow cytometry plots showing CD303 expression using a REAfinity anti-CD303 antibody conjugated to PE-Vio770 (top) or the previously used antibody clone conjugated to BV421 (bottom). **(b)** Bar graphs indicating the percentage of CD303+ cells (left) and MFI (right) for the PE-Vio770-conjugated antibody under three blocking conditions: human IgG alone, TruStain alone, or a combination of human IgG and the monocyte blocker True-Stain. **(c)** Bar graphs showing the percentage of CD303+ cells (left) and MFI (right) for the BV421-conjugated antibody under the same blocking conditions.
